# Machine learning of reverse transcription signatures of variegated polymerases allows mapping and discrimination of methylated purines in limited transcriptomes

**DOI:** 10.1093/nar/gkaa113

**Published:** 2020-02-25

**Authors:** Stephan Werner, Lukas Schmidt, Virginie Marchand, Thomas Kemmer, Christoph Falschlunger, Maksim V Sednev, Guillaume Bec, Eric Ennifar, Claudia Höbartner, Ronald Micura, Yuri Motorin, Andreas Hildebrandt, Mark Helm

**Affiliations:** 1 Institute of Pharmaceutical and Biomedical Sciences, Johannes Gutenberg-University Mainz, Staudingerweg 5, 55128 Mainz, Germany; 2 Epitranscriptomics and Sequencing (EpiRNA-Seq) Core Facility, UMS2008 IBSLor CNRS-Université de Lorraine-INSERM, Biopôle, 9 Avenue de la Forêt de Haye, 54505 Vandœuvre-lès-Nancy, France; 3 Institute of Computer Science, Johannes Gutenberg-University Mainz, Staudingerweg 9, 55128 Mainz, Germany; 4 Department of Organic Chemistry, Leopold Franzens University Innsbruck, Innrain 80/82, 6020 Innsbruck, Austria; 5 Institute of Organic Chemistry, Julius Maximilian University Würzburg, Am Hubland, 97074 Würzburg, Germany; 6 IBMC, UPR9002 CNRS-Université de Strasbourg, 2 Allée Konrad Roentgen, 67084 Strasbourg, France; 7 IMoPA, UMR7365 CNRS-Université de Lorraine, Biopôle, 9 Avenue de la Forêt de Haye, 54505 Vandœuvre-lès-Nancy, France

## Abstract

Reverse transcription (RT) of RNA templates containing RNA modifications leads to synthesis of cDNA containing information on the modification in the form of misincorporation, arrest, or nucleotide skipping events. A compilation of such events from multiple cDNAs represents an RT-signature that is typical for a given modification, but, as we show here, depends also on the reverse transcriptase enzyme. A comparison of 13 different enzymes revealed a range of RT-signatures, with individual enzymes exhibiting average arrest rates between 20 and 75%, as well as average misincorporation rates between 30 and 75% in the read-through cDNA. Using RT-signatures from individual enzymes to train a random forest model as a machine learning regimen for prediction of modifications, we found strongly variegated success rates for the prediction of methylated purines, as exemplified with *N*^1^-methyladenosine (m^1^A). Among the 13 enzymes, a correlation was found between read length, misincorporation, and prediction success. Inversely, low average read length was correlated to high arrest rate and lower prediction success. The three most successful polymerases were then applied to the characterization of RT-signatures of other methylated purines. Guanosines featuring methyl groups on the Watson-Crick face were identified with high confidence, but discrimination between m^1^G and m^2^_2_G was only partially successful. In summary, the results suggest that, given sufficient coverage and a set of specifically optimized reaction conditions for reverse transcription, all RNA modifications that impede Watson-Crick bonds can be distinguished by their RT-signature.

## INTRODUCTION

With the discovery of retroviral reverse transcriptases (RT) in 1970 by Howard Temin (1) and David Baltimore (2), the possibility of synthesizing cDNA copies of RNA substrates revolutionized the field of molecular biology and found application in various analytical and biotechnological methodologies, including transcriptome profiling, RNA structure mapping, RT-PCR of RNA fragments and RNA-sequencing (RNA-Seq) (3,4).

The most widely applied reverse transcriptases derive from the Avian Myeloblastosis Virus (AMV) and the Moloney Murine Leukemia Virus (MMLV), (5). In general, known reverse transcriptases exhibit two catalytic activities: a DNA polymerase activity and an associated RNase activity. Thereby, the DNA polymerase activity is used to copy both, RNA and DNA templates and the RNase activity, termed RNase H, degrades RNA in RNA–DNA-hybrid duplexes. The mature enzymes are formed by proteolytic processing and differ in their subunit structures. AMV RT is a heterodimer in which both subunits have DNA polymerase and RNase H activity, while MMLV RT is a monomer, that also exhibits both activities (6).

Today's commercially available reverse transcriptases are generally recombinant variants of the native AMV and MMLV enzymes with improved fidelity, elevated temperature optima, reduced RNase H activity and higher processivity, thus addressing limitations such as impairment through higher-order RNA structures, primer mispairing or relatively high error rates (7). Another well-known and researched reverse transcriptase is the heterodimeric HIV-RT, comprised of two related subunits. The larger subunit p66, carrying the polymerase and RNase H domains, plays the catalytic role, the smaller subunit p51 plays a structural role (8). HIV-RT serves as major target for antiretroviral drug combination therapies and therefore is mostly used in terms of drug development studies. Recently, HIV-RT was used in an evolution platform to find a reverse transcriptase variant with robust read-through and high mutation rates at modified sites during reverse transcription, allowing site-specific identification (9). There are also reports of increased reverse transcriptase activity from some DNA polymerases like Thermus thermophilus (Tth) (10), and a recent study about a N-terminally shortened form of the DNA polymerase I from Thermus aquaticus (Klentaq) (11) that shows the possibility of targeted polymerase engineering to evolve reverse transcriptase activity (12,13). In follow-up studies, Klentaq variants for specific modification analysis could be developed (14,15).

Until the development of nanopore-based direct RNA-Sequencing methods (16–19), reverse transcription of RNA templates was a mandatory step in library preparation for RNA-Sequencing (RNA-Seq), a widely used application for RNA sequence elucidation, including the detection of RNA modifications, the so-called modification calling (20). Thereby, the information about modification type and position may get erased, partially or completely, since the newly synthesized cDNA is composed only of the four canonical deoxynucleotides.

Of the >150 different types of post-transcriptional modifications (21) only a very limited number can alter cDNA synthesis due to chemical alterations on their Watson–Crick face and therefore leave traces in RNA-Seq data. The emerging characteristic patterns, which we previously termed RT-signatures, derive from RT termination products and misincorporated nucleotides, so-called mismatches, at modified sites and can be utilized to accurately detect RNA modifications, successfully applied on m^1^A (22). The latter, a modification featuring a methyl group on the Watson-Crick face of adenosine, which interferes with proper base-pairing, in RNA structure (23–25) as well as during cDNA synthesis by reverse transcription (26).

Two studies for a transcriptome-wide m^1^A detection based on the enrichment of m^1^A-containing RNA fragments with specific m^1^A antibodies and following mapping at single-nucleotide resolution were published in 2017 (27,28). By relying on the property of m^1^A to lead to typical misincorporation and truncation profiles by using different reverse transcriptases, this led to quite different outcomes in terms of the number of detected and proposed m^1^A sites. A recently published study on the cross-reactivity of the most commonly used m^1^A-binding antibody in transcriptome-wide mapping studies provides a possible explanation for the strongly varying numbers of m^1^A sites (29,30). Besides this, the two methods differ in their experimental design and the bioinformatic processing pipeline, but both use the thermostable group II intron reverse transcriptase (TGIRT) for cDNA synthesis. This enzyme is encoded by Group II introns, mobile ribozymes that use the RT to stabilize the RNA structure for splicing, and afterwards convert the integrated intron RNA back into DNA (31). The same reverse transcriptase was used for a study about high-throughput tRNA sequencing, where the authors focused on *N*^3^-methylcytidine (m^3^C) and *N*^1^-methylguanosine (m^1^G), two other modifications with altered Watson–Crick edge, among m^1^A (32). In addition, the occurrence of RT-signatures at two *N*^6^,*N*^6^-dimethyladenosine (m^6^_2_A) residues in yeast 18S rRNA is also known and reported to be inconsistent and partially indistinguishable from the signature of m^1^A (22,33). This raises the question whether purine modifications like m^1^G, m^2^G, m^2^_2_G, m^1^A, m^6^A or m^6^_2_A with altered Watson-Crick-Face show characteristic and distinguishable RT-signatures in RNA-Seq data and furthermore whether they can be clearly detected using computer-based analytical methods.

Central and common problems in modification calling from RNA-Seq data are rooted in statistics and data treatment. As outlined above, interpretation of RNA-Seq datasets is frequently controversial, as different groups apply variegated statistical methods, but also because, in the absence of commonly accepted standards, many thresholds set for modification calling are set arbitrarily, or according to idiosyncratic reasoning. Consequently, there is a need to establish neutral and unbiased procedures for threshold determination. Indeed, a number of bioinformatics tools can be applied to assess the robustness and accuracy of the modification calling based on RT-signatures. This concerns in particular the distinctness of actual modification sites from non-modified sites in RNA-Seq data. For this purpose, prediction models from machine learning are used, which can be implemented for classification and regression analysis. The use of machine learning models, trained to distinguish a certain class by different characteristics, allows a prediction without thresholds or cutoffs set by the user. The decision whether a modification is present or not is entirely left to the model, making the analyses comparable and less bias susceptible, as the model compromises between sensitivity (recall) and precision, while own thresholds and cutoffs tend to shift this balance to one side. Two examples of such supervised learning models for pattern recognition are support-vector machines (SVM) (34) and random forests (RF) (35), commonly used for binary classification. Random forest models were chosen as prediction tool in the study by Hauenschild *et al.* (22) operating by constructing decision trees, the building blocks of random forests, with training datasets and then being applied on test data for m^1^A recognition, according to the occurring RT-signatures. Random forests consist of a large number of individual decision trees, trained with different subsets of the training data, and each node of each decision tree being split using the feature with the best splitting properties (out of a random feature sampling) from the data. In operating as an ensemble, all prediction trees contribute to the overall classification, forming the model's prediction by majority voting (bagging) (35). The identification of the predictive performance and statistical classification is of particular importance in order to classify the received results. Therefore, a confusion matrix is created to allow visualization of the performance of a classification due to the algorithm (36). A confusion matrix consists of two rows and two columns reporting the number of false positives (FP), false negatives (FN), true positives (TP) and true negatives (TN), comparing the instances in a predicted class with the instances in an actual class. For more detailed analysis, the calculation of the sensitivity, also called true positive rate (TPR), the specificity, true negative rate (TNR), or precision, positive predictive value (PPV), is possible. By plotting the true positive rate against the false positive rate (FPR), calculated as (1 − specificity), a receiver operating characteristic (ROC) curve as two-dimensional graphical plot can be generated to illustrate the classifier performance (37). For comparison purposes, the area under the ROC curve (AUC) as scalar value is a good measure for the classifier performance (38,39). Another quality parameter is the Matthews Correlation Coefficient (MCC), which serves to study the correlation between observed and predicted binary classifications and can be used even if the classes are of very different sizes (40).

Here, we present an RT-signature analysis of various purine modifications, featuring methyl groups on the Watson–Crick face known to impede reverse transcription. The analyses uncovered a strong dependence of RT-signatures on the reverse transcriptase enzyme, and, most interestingly, a correlation between different RT-signature characteristics and success rates of random forest models for prediction of modified sites. Different reverse transcriptase enzymes produce substantial differences in mismatch, arrest and read-through feature content and the variegated RT-signatures lead to highly variable machine learning performances. As exemplified with *N*^1^-methyladenosine (m^1^A), the reverse transcriptase selection can strongly change and increase the detection rates, by prediction, based on different importance of the characteristics. These findings help to enable a more precise detection of modified sites, by selecting the best enzymes and using a set of specifically optimized reaction conditions, and can therefore be used for future modification studies.

## MATERIALS AND METHODS

The synthetic nucleic acids for purine modification analysis (m^6^A, m^6^_2_A, m^1^G and m^2^_2_G) were synthesized according to previously published protocols (41,42). The m^1^A oligonucleotides and all other synthetic nucleic acids were from IBA (Göttingen, Germany). Details including sequence information are given in Supplement Table S1. For the studies, total tRNA from *Saccharomyces cerevisiae* from Roche was used (Sigma-Aldrich, Germany). The amount and quality of the RNA was verified by UV absorption (NanoDrop 2000 (Thermo Fisher Scientific, Germany)), polyacrylamide gel electrophoresis (PAGE) and Agilent RNA ScreenTape analysis (for Agilent TapeStation 4200).

### Library preparation & sequencing

Library preparation was prepared according to a previously published version (22,43) with necessary adaptations for the reverse transcription and 3′-tailing step (details including sequence information of the used synthetic nucleic acids are given in Supplement Table S1). Briefly, total tRNA from *Saccharomyces cerevisiae* and the synthetic revolver oligos (the corresponding m^1^A, m^1^G and m^2^_2_G revolver oligos as synthesized, and the m^6^A and m^6^_2_A revolver oligos mixed in equal amounts respectively) were dephosphorylated with FastAP Thermosensitive Alkaline Phosphatase (Thermo Fisher Scientific, USA) as previously described. Then, a pre-adenylated 3′-adapter was ligated to the 3′- end of the dephosphorylated RNA using T4 RNA ligase 2 truncated (New England Biolabs, Germany) and T4 RNA ligase (Thermo Fisher Scientific, USA). After purification with 5′-Deadenylase (New England Biolabs, Germany) and Lambda exonuclease (Thermo Fisher Scientific, USA) to remove non-ligated pre-adenylated adapter, an ethanol precipitation was performed. The RNA pellet was re-dissolved and used for reverse transcription. The reverse transcription step was performed with slight adaptation of the previously published workflow as follows:

*Reverse transcription*. For the reverse transcription step, we used 13 different reverse transcriptases (see Table 1) and the reaction was performed according to the respective manufacturer's protocol (see Supplement Table S2). In general, the reverse transcription mixture (20 μl) comprised the RT primer (IBA, Germany; see Supplement Table S1 for sequence) in a final concentration of 5 μM and the respective RT reaction buffer (1×). The mixture was heated to 75°C for 5 min for heat denaturation, then chilled on ice. Then, dNTP mix (0.5 mM final concentration) and according to the protocol BSA, DTT and/or MgCl_2_ (see Supplement Table S2) were added to the mixture. After addition of the RT enzyme, the reaction was performed at 45°C for 1 h with one exception, for the DNA polymerase variant Volcano (RT #13) the reaction was done at 60°C for 1 h.

**Table 1.** Reverse transcriptases (RTs)

	Reverse transcriptase	Origin	Cat. No.	Supplier
**#1**	**M-MuLV**	MMLV	M0253S	New England Biolabs
**#2**	**AMV**	AMV	M0277S	New England Biolabs
**#3**	**ProtoScript^®^ II**	MMLV	M0368S	New England Biolabs
**#4**	**GoScript™**	MMLV	A5003	Promega
**#5**	**SuperScript™ III**	MMLV	18080044	Thermo Fisher Scientific
**#6**	**RevertAid™**	MMLV	EP0441	Thermo Fisher Scientific
**#7**	**AccuScript™**	MMLV	600089	Agilent
**#8**	**AffinityScript™**	MMLV	600107	Agilent
**#9**	**M-MLV**	MMLV	M1701	Promega
**#10**	**MonsterScript™**	MMLV	MSTA5110	Epicentre^®^
**#11**	**EpiScript™**	MMLV	ERT12910K	Epicentre^®^
**#12**	**SuperScript™ IV**	MMLV	18090050	Thermo Fisher Scientific
**#13**	**Volcano**	Klentaq	#8100S	myPOLS Biotec

After reverse transcription, excess of the RT primer was digested using Lambda exonuclease (Thermo Fisher Scientific, USA) and Exonuclease I (Thermo Fisher Scientific, USA). Residual dNTPs were dephosphorylated with FastAP Thermosensitive Alkaline Phosphatase (Thermo Fisher Scientific, USA). Next, RNA was degraded by addition of NaOH, the sample was neutralized with acetic acid and the cDNA was ethanol precipitated. The 3′-tailing and ligation step of the cDNA were performed with slight adaptations to the previously published workflow as follows:

*3′-Tailing and ligation of cDNA*. For the purine modification studies, adenosine triphosphate (ATP) was used in the tailing step and a 5′-adapter with TT sequence overhang (5′-adapter strand 2) was used accordingly to create the double-stranded DNA adapter for the second adapter ligation. After re-dissolving the cDNA pellet, the 3′-cDNA tailing was performed using the enzyme terminal deoxynucleotidyl transferase (TdT) (Thermo Fisher Scientific, USA) in 10 μl reaction volume. The reaction mixture included TdT Buffer (1×), rATP (final concentration: 1.25 mM) and TdT (final concentration: 1 U/μl). The double-stranded DNA adapter was prepared by annealing equimolar amounts of the synthetic single-stranded oligonucleotides 5′-adapter strand 2 & 5′-adapter strand 3 (see Supplement Table S1 for sequences) at 90°C for 5 min and subsequent cooling to 22°C and further incubation for 10 min (Cytidine triphosphate (CTP), 5′-adapter strand 1 with GG sequence overhang & 5′-adapter strand 3 were used for the side-by-side m^1^A RT-signature study according to the previously published protocol (43)). The ligation of the double-stranded DNA adapter (final concentration 1.25 μM) was then performed overnight at 4°C in a total volume of 40 μl with T4 DNA ligase HC 30 U/μl (Thermo Fisher Scientific, USA) and ATP (Thermo Fisher Scientific, USA) in a final concentration of 1.5 Weiss U/μl DNA ligase and 10 μM ATP in 50 mM Tris–HCl (pH 7.4) and 20 mM MgCl_2_ reaction buffer. Ligated DNA was then ethanol precipitated, and the ligation product was loaded on a 10% denaturing polyacrylamide gel and size selection between 40 and 150 nt was performed to purify ligated DNA from non-ligated DNA adapters.

After elution and ethanol precipitation the samples were used for a final PCR amplification with Taq-Polymerase (Rapidozym, Germany) and P7 and P5 PCR primers. PCR products were ethanol precipitated and loaded on a 10% denaturing polyacrylamide gel for size selection between 150 and 300 nt. After elution, ethanol precipitation and re-dissolving, the libraries were loaded on an Agilent High Sensitivity DNA chip (for Agilent Bioanalyzer 2100) to check for the presence of adapter dimers. Each library was then quantified by fluorometry using the Qubit dsDNA HS assay kit (Thermo Fischer Scientific, USA) and finally sequenced after pooling on MiSeq in 2 × 75 bp paired-end mode. Demultiplexing was performed and the resulting FastQ files were inspected for a quality check using FastQC.

### Data analysis

The sequencing data was processed using multiple bioinformatic analysis workflows. These workflows entail functionalities for standard procedures such as adapter trimming and reference mapping, but also provide algorithms for the extraction of relevant information from the alignment data, automated machine learning and prediction for modification calling as well as for visualization, based on our previously published software package (44). Described below are the workflows used in context of this study. As of note, these workflows were integrated into the graphical user-interface Galaxy which provides easy accessibility, adjustability and reproducibility (45). A version of said Galaxy-platform including many of the workflows used in this study is available for download and is described in Schmidt *et al.* (46).

### Standard workflow

The standard workflow performs automated trimming, mapping and feature extraction and was used to analyze all datasets. In general, the workflow is derived and adapted from Hauenschild *et al.* (44) and implemented in Galaxy.

#### Trimming

In the first trimming step, auxiliary sequences such as adapters, unique molecule identifiers (UMIs) and tailing-bases were removed from the reads using the Cutadapt (version 1.16) software (47). Due to the specifications of the library preparation, this step was performed separately for forward and reverse reads as they require different settings. The trimming step consisted of two phases: Removal of the Illumina adapters and the trimming of the UMI sequences, 10 bases (nine of them random) located at leftmost (forward read) or rightmost position (reverse read). In addition, the remaining sequences were filtered by length with the required minimum length being set to 10.

#### Alignment

Mapping of the reads to the reference sequence was performed using the Bowtie2 alignment software (48). As in the trimming step, mapping was applied separately for forward and reverse reads. The settings for Bowtie2 allowed for one mismatch (‘-N 1’-option) in a sequence of six bases (‘-L 6’) to account for the high number of expected mismatches from remaining tailing bases (added in an enzymatic step with TdT during library preparation at the 3′-end of the cDNA as DNA adapter anchor for the adapter sequence overhang; excess possible, therefore additional overhang trimming step in post-processing after alignment) and high amounts of modified ribonucleotides contained in the underlying tRNA samples. The two alignment files from forward and reverse reads were stored in BAM-format and then merged into a single file. The BAM file were then sorted and indexed using the SAMtools package (49).

#### Post-processing and feature extraction

The alignment data was converted into pileup format as this format allows to extract relevant information for each position in the reference. Remaining tailing bases were removed from the alignment using a custom python script (overhang trimming). The format was then converted to a tab-separated text-file termed ‘Profile’, containing information on multiple defined features such as the coverage, arrest rate, mismatch rate, the mismatch composition and the jump rate for each individual position within the reference. The features are defined as follows:

**Coverage**: Describes the number of reads/nucleobases that were mapped to the position in the reference.
**Arrest rate**: The arrest rate of a given position *x* is defined by the relative number of reads that start at the neighboring position *x* + 1. Since the arrest is given as a percentage in dependence of the coverage, it can also be described as the drop in coverage in relation to the preceding position.
**Mismatch rate and mismatch composition (**individual misincorporation rates): The mismatch rate is the relative number of nucleobases mapped to a given position that do not match to the nucleobase shown in the reference. The Profile does not only contain the mismatch rate, but also gives detailed information on the alignment numbers for each type of base (A, C, G, T) and unknown read bases (N). For each individual nucleobase, the amount of mismatch relative to the overall mismatch is also calculated, thus resulting in values for A-, C-, G- and T-mismatch.
**Jump rate**: A jump is represented by deletions occurring at the position in question or shortly thereafter. Jumps are presented as percentage of deletions relative to the overall coverage.

### Machine learning and prediction

In order to determine whether a given position in the alignment is modified or not, a machine learning algorithm based on a random forest (RF) model was used (35). Our supervised prediction workflow is thereby modeled according to our earlier study on m^1^A RT-signatures from Hauenschild *et al.* (22,43), to allow comparison. Here, we used the random forest model integrated into the Python ‘scikit-learn’ package (version 0.20.2) (50). In general, random forest models generate a multitude of decision trees based on the available training data by separating instances from two predefined and known classes (positive and negative class) according to a number of features describing each instance (mismatch rate, arrest rate, jumps, etc.). For the prediction process (e.g. classifying an instance of which the class is not known), an instance is put through every individual decision tree where it is eventually assigned a class. The instance is then classified according to the ‘majority vote’ of all individual decision trees.

For the special properties of this study, such as the number of modified instances being generally quite low compared to non-modified instances, the model had to be adjusted to accommodate this problem. As a first measure, for the training process, the machine learning model was given positive and negative classes (e.g. modified and unmodified instances) in a ratio of 1:1. Secondly, the model was enhanced by *k*-fold stratified cross-validation. This approach splits the available training dataset into *k* subsets. The random forest is then trained on *k* − 1 subsets and performs a prediction on the remaining subset. The whole process is performed *k* times with a different subset being left out in each iteration. In our studies, we set *k* to 5. With this approach, the random forest model makes use of all available data as opposed to simply splitting the data into training and test samples as the test sample would not be used for training. Accordingly, cross-validation is especially useful for datasets of low sample sizes. To counteract any possible biases resulting from the 1:1-ratio of modified and unmodified instances and the thereof resulting reduction of the number of unmodified instances considered in the training process, the whole cross-validation process was repeated 10 times. In each iteration, the unmodified instances were randomly selected.

This approach not only generated a trained random forest as outcome, but also allowed the evaluation of the model's predictive accuracy. Assessment scores for the random forest performance such as the AUC, MCC, sensitivity and specificity were calculated and averaged over the 10 iterations of the training process.

For the prediction process in this study, we trained a random forest model based on the instances of a specific modification from two independent tRNA datasets and subsequently performed classification on a third tRNA dataset.

## RESULTS

### Side-by-side comparison of 13 RT-signatures at known m^1^A sites

To assess the influence of the enzyme on RT-signatures, we used the previously established workflow to detect variations in m^1^A-related signatures of a total of 13 enzymes (see Table 1). These 13 reverse transcriptases represent a selection of commercially available RTs at the beginning of the study (without claiming completeness). Briefly, yeast total tRNA was submitted to an RNA-Seq based on a library preparation protocol designed to capture misincorporation events as well as abortive cDNA (43). The reads were mapped onto a reference set consisting of yeast tRNA sequences, compiled from the Modomics (21) and tRNA database (51), containing sequences of known modification status.

**Figure 1.**
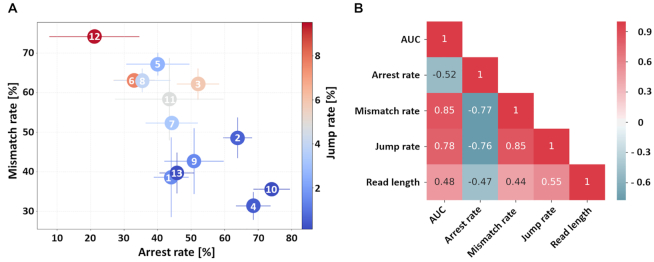
(**A**) Scatter plot showing the average m^1^A signatures of 13 RTs at 26 m^1^A sites in yeast cytosolic tRNA. Error bars show standard deviations of arrest and mismatch rates across 3 sequencing runs, i.e. triplicates. The colour-code represents the jump rate. Arrest rate percentages refer to the reads covering the 3′ adjacent position of m^1^A (+1 position). Mismatch and jump rate percentages refer to the reads covering the m^1^A position. (**B**) Pearson correlation coefficient (PCC) matrix heat map. Evaluation of interrelations and mutual influences by showing the positive and negative correlations between the RT-signature features arrest, mismatch and jump rate as well as the random forest performance measure AUC (Area Under Curve) and the read length (see also Supplement Figure S9 for additional information on TGIRT and HIV-RT).

From all m^1^A sites listed in Modomics and the tRNA database, those were excluded from further analysis, for which the coverage was insufficient to extract a statistically relevant RT-signature. At m^1^A sites, corresponding signatures were extracted which are composed of the following features: (i) the overall mismatch incorporation of nucleotides into the cDNA, (ii) the individual mismatch compositions (plotted in Supplement Figure S1) and (iii) coverage drops between m^1^A and the position to its 3′ due to RT-arrests. In addition to these previously (22) established features, (iv) a new feature termed ‘jump rate’ was established. This refers to a certain amount of deletions in the aligned data, specifically detected at m^1^A sites. Such jumps are known from DNA polymerases, proposed mechanisms include strand slippage (52,53) and strand misalignments during DNA synthesis (54–56). A comprehensive illustration and the data of the distribution of RT-signature features (i, iii and iv) in the different enzymes at m^1^A sites is shown in Figure 1A and Supplement Figure S2. Of note, all used datasets contained at least 300 000 reads per RT-enzyme (per replicate), and escalating comparative analyses with smaller datasets indicated no significant changes even at read numbers as low as 50 000 reads (see Supplement Figure S10). From Figure 1A, it is apparent that the 13 reverse transcriptases display a wide distribution of features i, iii and iv, which is bracketed between RT #12, with highest mismatch, highest jump rate and lowest arrest rate on one hand, and RTs #4 and #10, with lowest mismatch rates, lowest jump rate and highest arrest rate, on the other hand. A distribution along the diagonal, which is immediately obvious upon visual inspection, strongly suggests an inverse correlation of arrest and mismatch rates, and indeed, the corresponding Pearson correlation coefficient (PCC) is −0.77. Further correlations, also including the overall average read lengths (see Supplement Figure S3), are shown in the correlation matrix in Figure 1B.

Interestingly, the jump rate shows a positive PCC with the mismatch rate (0.85), and consequently a negative correlation (−0.76) with the arrest rate. On the molecular level, this suggests a mechanistic relation between a generally high elongation activity of a given enzyme on one hand, and successful bypassing of m^1^A sites, which is reflected in low arrest rates and correspondingly higher jump and misincorporation rates, as well as in the corresponding correlations with the average read length.

### Random forest performance and feature importance of individual RTs

Based on the known m^1^A sites in yeast tRNA, the signatures with their characteristic features were used as input for machine learning analysis. As machine learning model, a random forest implementation was trained on m^1^A and non-m^1^A instances to perform binary classification, and thus conduct modification calling without external input of threshold values. The quality of the classification was compared among all 13 RTs by determining the area-under-the-curve value (AUC) of a receiver operating characteristic (ROC) curve. The calculated AUC results from a machine learning approach applying 10 repetitions of a 5-fold cross validation.

AUC values are frequently used for such quality assessments, as they provide a measure of the balance between sensitivity and specificity instances in modification calling. To safeguard against inadverted optimization by the random forest model of parameters not reflected by the AUC, we also calculated the MCC (Matthews correlation coefficient) (40) as an additional quality parameter, which accounts for true and false positives and negatives and serves as correlation coefficient between observed and predicted binary classifications. In the remainder of the manuscript, we will report and discuss AUC values only, since the correlation between AUC and MCC results reported throughout this manuscript was 0.9016 (see Supplement Table S3).

**Figure 2.**
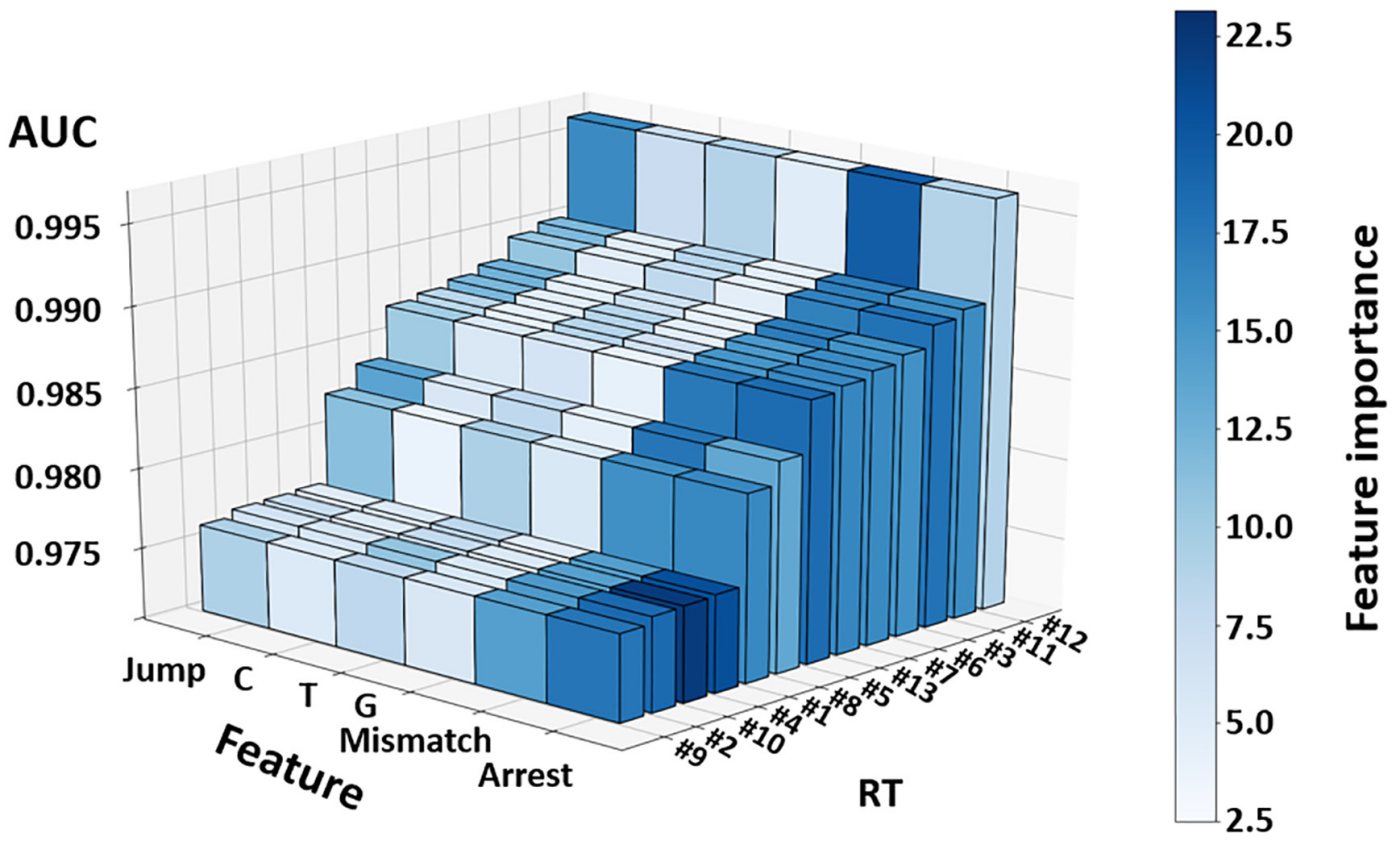
Bar plot with random forest performance and feature importance by RT. Classification performance is represented as Area Under Curve (AUC) of Receiver Operating Characteristic (ROC). Colour-code represents the feature importance for the classification. Data was averaged from triplicates. Jump = jump rate. C, T, G = mismatch components, which add up to 100%. Mismatch = mismatch rate. Arrest = arrest rate. Percentages represent feature importance in random forest analysis = mean loss in classification accuracy, if values of respective feature are permutated. (See also Supplement Figure S9 for additional information on TGIRT and HIV-RT)

Overall, AUCs for each individual RT varied substantially, in a range of 0.9764–0.9964, with the lowest AUC value registered for RT #9 and the highest recorded for RT #12 (see Figure 2 and Supplement Table S3). It is important to stress, that even variations at the third and fourth significant digit are extremely relevant for modification calling; in an application of a trained random forest model to an epitranscriptome of 10^7^ adenosines, the difference between 0.998 and 0.999 is projected to cause an additional 10^4^ errors. We therefore inspected the features of the random forest in some detail. Figure 2 and Supplement Table S4 show the feature importance, i.e. the relevance of the individual feature for the decision making in the training process. These include the previously discussed features (i–iv), with the individual misincorporation rates (ii) separated according to the three nucleotides (iiC, iiT, iiG). The variegated levels of feature importance, represented as blue shades, provides interesting insight into the ‘strategy’, by which different RTs (in combination with the corresponding random forest) arrive at optimum prediction performance. This analysis of the feature importance (indicated by a color shade code) provides a basis to support the intuitive (and hence anticipated) claim that certain polymerases allow detection of m^1^A (and probably other modifications) via RT-arrest, while others provide significant information in the form of misincorporation. More specific is the finding that an increasing feature importance of the jump rate (iv), correlates with a decline of importance of the arrest rate (iii). This was most notable for RT #12 which was the one RT standing out because of its very low importance of the arrest rate, contrasted by high importance of jump and mismatch features. Indeed, higher arrest rates were found to be associated with lower AUC values in general, indicated by a negative PCC (−0.52) between the two values (see Figure 1B). A plausible explanation might be the low coverage generally associated with RT-enzymes of high arrest rate, which, in turn, renders the sites toward the 5′-end, less amenable to statistically significant analysis.

In contrast to the arrest rate, the correlation for mismatch and jump rates with the AUC was positive (0.85 and 0.78). Feature importance of the individual mismatches (C, T and G mismatch) were very low to negligible for C and G mismatch. Solely T mismatch levels played a role in the machine learning process as is already implied by the prominent levels of T mismatch at m^1^A positions (≈50%) (plotted in Supplement Figure S1).

While the above considerations result from perusal of the ensemble of polymerases, a closer look at the features of the top three performing enzymes revealed interesting details with potential impact for prediction quality. RT #11 for example, despite being rather processive, uses arrest events to almost equal degree (15.952%; dark blue colour in Figure 2) than it does mismatches (17.112%), whereas RT #3 also uses both features about equally (arrest 18.253% and mismatch 16.933%) but with slight preference for arrest. RT #12 finally is the most processive among all RTs, which presumably leads to a high percentage of jumps as already pointed out (see Figure 1A). The latter feature is exploited by the random forest, which heavily relies on jumps (16.020%) for modification calling (see Figure 2 and Supplement Table S4). In summary, and against the general trend that associates success with processivity, the three best enzymes according to their individual random forest performance use three different strategies for optimal modification calling results. We therefore selected these polymerases with their different characteristics, and compared their behaviour on other purine modifications as well (*vide infra*). To guard against a training bias potentially originating from the use of m^1^A instances in the structural context of a tRNA, we performed experiments using synthetic m^1^A-containing oligos (Supplement Table S9) designed according to the ‘revolver’ concept, i.e. with a degenerated nucleotide at the +1 position to ablate the previously identified influence of this position on mismatch composition (22). Performance parameters obtained from random forests trained with 26 native m^1^A sites were indistinguishable from those trained with an additional 56 synthetic sites (Supplement Table S10), suggesting that the results were not impacted by any potential overfitting.

To account for other m^1^A detection data, meanwhile published during or after our study, mainly based on two other reverse transcriptases, TGIRT (27,28) and HIV-RT (9), we retrospectively collected data on m^1^A RT-signatures and the m^1^A random forest performance for these RTs to allow evaluation and comparison (see Supplement Figure S9 for experimental and analytical details). The reverse transcriptases were subjected to the same library preparation and data analysis workflow, whereby for the TGIRT the manufacturer's (RT #14a) (as applied in (28)) as well as an adapted buffer composition (as applied in (27)) were tested. The adapted TGIRT (RT #14b), as well as the wildtype HIV-RT (RT #15) led to mismatch and arrest patterns at m^1^A sites which were in the range of RT #12, lacking the distinct jump characteristics. In comparison, RT #14a provides RT-signatures with comparable mismatch rates, also lacking increased jump rates, but with much higher arrest rates (see Supplement Figure S9A and B). The average read length provided by RT #14b exceeds the previous values of all other RTs, indicating a high read-through capability. RT #15 and RT #14a are in line with the values of the other RTs. In terms of machine learning performance, RT #14b achieves an AUC in the range of the best performing RT #12, while RT #14a and RT #15 do not reach the AUC values of the best RTs (see Supplement Figure S9D). The behaviour of these RTs with respect to feature importance in machine learning corresponds to the findings of our previous RT analysis, so that more distinct features lead to stronger feature importance in random forest models, while weak or missing features play only a minor role.

### Machine learning for modified guanosines

**Figure 3.**
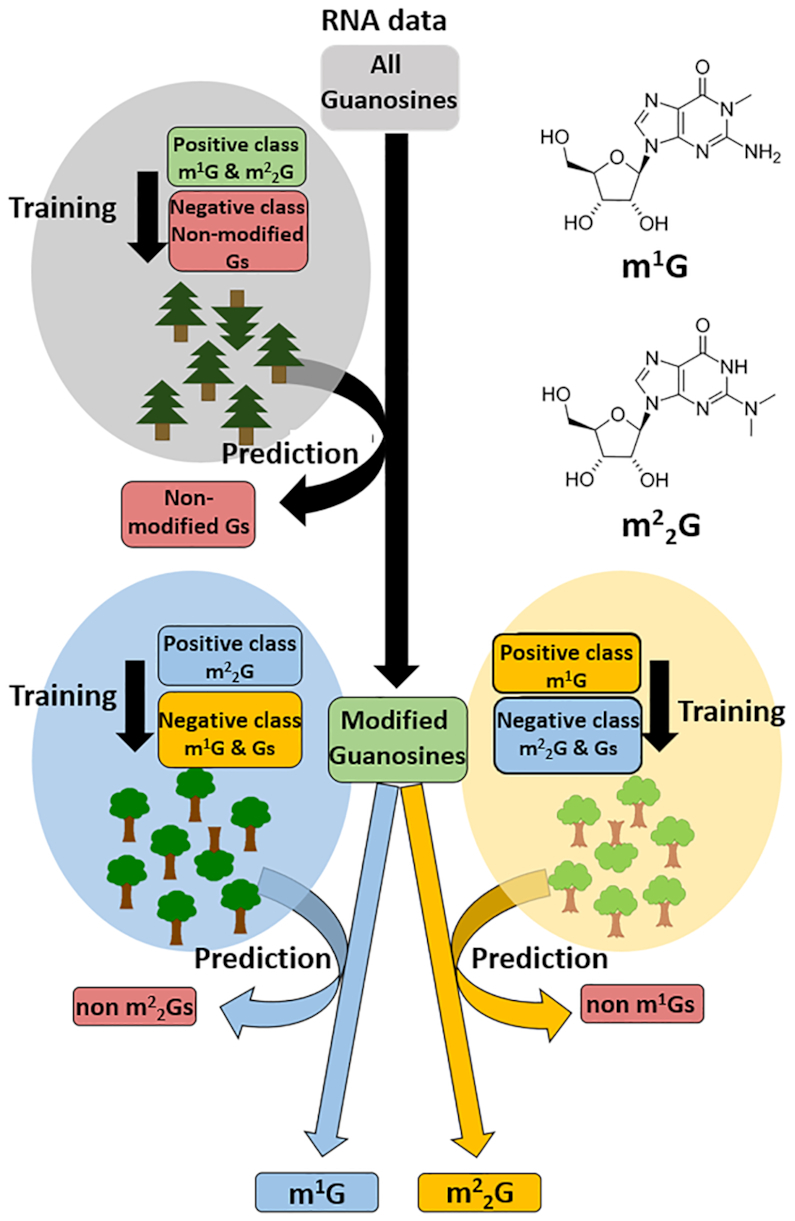
Prediction scheme for guanosine modifications. A first random forest model was trained (10-repetitions 5-fold cross validation) separately by RT on m^1^G & m^2^_2_G sites from two replicates of total tRNA samples from *Saccharomyces cerevisiae* (RNA data) to distinguish modified (positive class) from unmodified (negative class) guanosines. The trained random forest then was used to make a prediction on guanosines from a corresponding third yeast data set. In this case, the prediction is a binary classification, the two classes being ‘m^1^G, m^2^_2_G’ or ‘non-modified Gs’. Guanosines which were classified as modified guanosines (m^1^G and m^2^_2_G) in the first prediction were then written into an output file (RNA data modified Gs). Then, two random forest models were trained (10-repetitions 5-fold cross validation) separately by RT on either m^1^G or m^2^_2_G sites from two replicates of total tRNA samples from *S. cerevisiae* (RNA data) as positive class to separate these guanosine modifications from each other (the respective other modification together with non-modified Gs (1:1 ratio) served as negative class). The trained models were then used to make a prediction on the output file from the first prediction (RNA data Modified Gs).

Similar to m^1^A, the methylated purines m^1^G and m^2^_2_G possess one methyl-group pointed towards the Watson–Crick face. The fact that m^1^G and m^2^_2_G are less abundant in tRNA than is m^1^A, presents a problem for valid training of a random forest model, which requires an adequate number of training instances for unbiased predictions. To increase the number of usable instances, we pooled m^1^G and m^2^_2_G sites in a first training run, which was designed to separate the modified guanosines from non-modified guanosines (see Figure 3 and Supplement Figure S4A). This left the option to distinguish between m^1^G and m^2^_2_G at identified sites later on, using divergence of particular features in their respective signatures. Analysis was conducted on RNA-Seq data from the three reverse transcriptases (#3, #11 and #12, as identified above) as well as from RT #5 for comparison with previous data (22). In full analogy to the previous evaluation of m^1^A sites, the corresponding reads were mapped onto a reference set consisting of tRNA sequences from *Saccharomyces cerevisiae*, compiled from the Modomics (21) and tRNA database (51). The data was inspected for signatures at known m^1^G and m^2^_2_G sites. We found, that many m^1^G and m^2^_2_G sites, which are typically present near the 5′-end at positions 9 and 26, displayed low coverage as a result of their location downstream of other RT-blocking modifications such as m^1^A or bulky modifications at position 34 and 37.

We prepared three independent RNA-Seq datasets from three replicates of total tRNA yeast for each of the four reverse transcriptases, each total tRNA dataset (each replicate) containing 988 guanosines, including 16 m^1^G and 22 m^2^_2_G sites. In general, two replicates were used for random forest training and the remaining third replicate for testing (RF #1). In order to collect a sufficient number of sites for random forest training, we set a relatively low coverage threshold of 20 reads. After removal of the low-coverage instances from the two training datasets, between 22 (68.8%) and 32 (100%) instances for m^1^G (dependent on the RT used; see Supplement Table S5) and between 20 (45.5%) and 38 (86.4%) instances for m^2^_2_G passed this coverage threshold (e.g. 32 m^1^G and 38 m^2^_2_G for RT #12, see Supplement Figure S4 (B)). Along with a matching number of unmodified guanosines as negatives, these were used for training a random forest model in full analogy to those used for m^1^A. In a first random forest model we trained and tested for a joint m^1^G and m^2^_2_G separation from other guanosines, so m^1^G and m^2^_2_G sites were together used for random forest training. Overall, the model prediction scored AUC values between 0.9439 and 0.9801 for the different RTs (e.g. 0.9801 for RT #12), which favorably compares to our first runs on m^1^A (22). After filtering to remove low coverage instances (coverage >20) we could correctly classify 80% of the m^1^G instances and 90% of the m^2^_2_G instances (average percentages for all RTs used) in the respective test datasets (see Supplement Table S5).

Besides m^1^G and m^2^_2_G other instances of Watson-Crick blocking modifications were classified as modified, filtering out non-modified guanosines in the process. An interesting instance of a ‘false’ positive concerned position 37 in yeast tRNA^Phe^, namely the wybutosine modification (see Supplement Figure S5). This hypermodification is generated in a sophisticated multistep biosynthesis starting with m^1^G at G_37_ (57). Clearly, the ‘Y-base’ features a blocked Watson–Crick face as well, and some similarities of its RT-signature to m^1^G and m^2^_2_G were to be expected. We conclude that purines with modifications blocking the Watson-Crick face can be successfully identified in a sequence space of 10^4^–10^5^.

In keeping with Figure 3 (see also Supplement Figure S4A) a second step (RF #2) was conducted with two independent random forests, which were separately trained on m^1^G and m^2^_2_G respectively. The respective other modification together with non-modified guanosines served as negative class (1:1 composition). The model predictions for m^1^G scored AUC values between 0.9226 and 0.9647 and AUC values between 0.9433 and 0.9820 for m^2^_2_G according to the different RTs (e.g. 0.9647 m^1^G and 0.9801 m^2^_2_G for RT #12). The trained models were then used in an attempt to distinguish the m^1^G vs. m^2^_2_G instances identified in the first step prediction and to reduce the number of false positives, so the trained random forest models were tested on all instances, classified as modified in the first step RF #1 (e.g. 67 instances for RT #12). In average, the four different RTs could identify and correctly classify 93.8% instances for m^1^G (see Supplement Table S6) and 88.7% instances for m^2^_2_G (see Supplement Table S7) from the previously classified modified sites (in RF #1) in the random forest models (see Supplement Figure S4B). With the second random forest model the number of false positives for all RTs could be reduced. Despite this, the resulting classification did not completely separate the two modified guanosines from each other and still includes a certain number of false positives for the respective modification. Even though in the case of RT #5 and #12 all m^1^G and m^2^_2_G sites from the first step were successfully retrieved and separated, the number of false positives still did not allow an accurate detection. Given that we had to set a relatively low coverage threshold, we wondered if low coverage might blur the characteristics in the RT-signatures to a degree that the random forest model suffers in prediction quality. Indeed, this is in line with an analysis of pseudouridine modification calling after CMCT treatment (58) and we thus hypothesized that the low coverage might be responsible for the lack of distinction between the RT-signatures of m^2^_2_G and m^1^G, respectively.

### Synthetic RNAs yield clear RT-signatures

**Figure 4.**
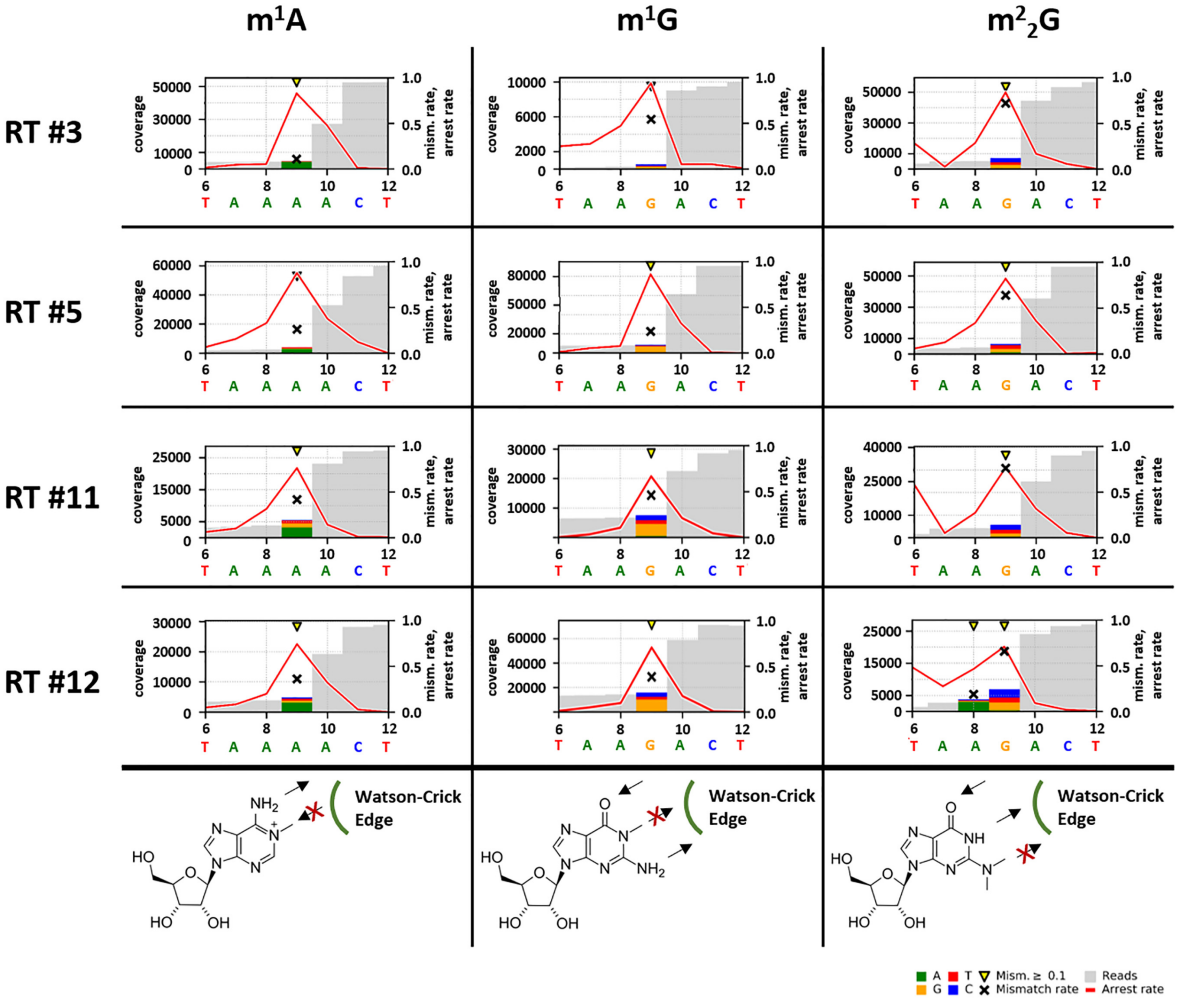
Examples for RT-signatures of m^1^A, m^1^G and m^2^_2_G by RT from revolver oligo analyses and the expected structural impairment of the Watson–Crick base-pairing. Graphs from the revolver oligo with a neighboring A, 3′ adjacent (+1 position) to the modified site at position 9, are shown. Sites with error rates of more than 10% are highlighted with yellow arrows. Colored bars indicate the nature of the reads. The mismatch rate is depicted as black cross and the arrest rate as red line. The modified site is shown at position 9 in the middle of the considered sequence. In general, arrest rate percentages refer to the reads covering the 3′ adjacent position of m^1^A/m^1^G/m^2^_2_G (+1 position). Mismatch rate percentages refer to the reads covering the modified position; Note that statements on average values stated in the text may differ from these individual signatures.

To verify this hypothesis, we synthesized and tested short oligoribonucleotides of a type previously published for m^1^A analyses (22). By analogy, these oligos are referred to as ‘revolver’ oligos according to the so-called revolver assay from our previous study (see Figure 3 in Hauenschild *et al.* (22)). They contained a central position occupied by the modified nucleotide under investigation, whereas the remaining sequence was identical. Because of the known influence of the neighbouring bases (+1 position, 3′ to the modification), four derivatives were designed and synthesized featuring all four different possible neighboring bases downstream of the respective modification. Figure 4 shows signals of the four enzymes, arranged for side-by-side comparison of m^1^A, m^1^G and m^2^_2_G. Data on m^6^_2_A, the fourth methylated purine with a blocked Watson−Crick edge, were omitted here, but are shown in the supplement (see Supplement Figure S6). In brief, our data for synthetic m^6^_2_A revolver oligos could confirm our previous observations at two residues in yeast 18S rRNA (22), showing inconsistent RT signatures. Therefore, modification calling based on machine learning models is not applicable and no further investigations of this modification were performed. In passing, we also verified that m^2^G and m^6^A did not produce any appreciable RT-signature (see Supplement Figure S7), which is in keeping with the methyl group pointing away from the Watson–Crick face during reverse transcription.

**Figure 5.**
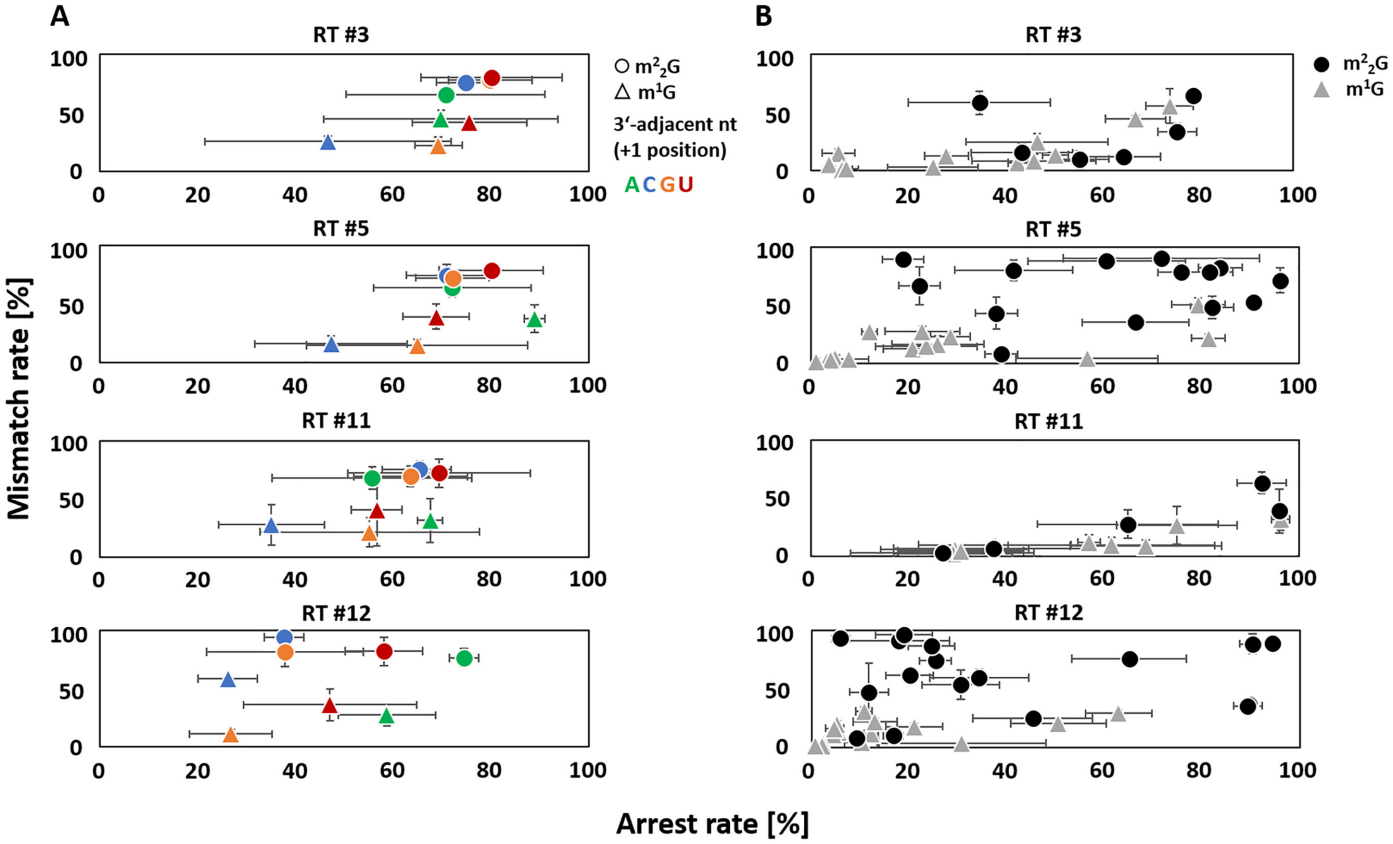
m^1^G and m^2^_2_G RT-signature comparison for the analysed reverse transcriptases RT #3, #5, #11 and #12. (**A**) Revolver oligo. Dot plots of mismatch and arrest RT-signatures at m^2^_2_G (dots) and m^1^G (triangles) sites in revolver oligos for base configurations guanosine (orange), cytidine (blue), uridine (red, T in mapping profile) and adenosine (green) at position +1. Mismatch and arrest rates are given in percentage. Data was averaged from triplicates; error bars show standard deviations of arrest and mismatch rates. (**B**) Total tRNA from Saccharomyces cerevisiae. Dot plots of mismatch and arrest RT-signatures at m^2^_2_G (black dots) and m^1^G (gray triangles) sites in total tRNA. Data was averaged from triplicates; error bars show standard deviations of arrest and mismatch rates. m^1^G and m^2^_2_G sites which are present in all three total tRNA replicates and show a coverage of at least 20 reads in at least two replicates are shown (see Supplement Table S8 for more details**)**. In general, arrest rate percentages refer to the reads covering the 3′ adjacent position of m^1^G/m^2^_2_G (+1 position). Mismatch rate percentages refer to the reads covering the modified position.

The two guanosine modifications m^1^G and m^2^_2_G produced consistent characteristic signatures for the different selected reverse transcriptases in the revolver oligo analyses. In general, the signatures for m^1^G and m^2^_2_G show high arrest rates, which were comparable to that of m^1^A. RT #3 and RT #5 featured the highest values, while the lowest values originated from the highly processive RT #12 (see Supplement Figure S8A). Interestingly, for each RT-enzyme, the mismatch rate at m^2^_2_G sites was higher than at m^1^G sites, such that one might anticipate a successful application of this feature in an random forest model (compare feature importance in Figure 2). A potential reason for the failure of our random forest approach became apparent upon comparison of the individual modified sites in native tRNA and in the synthetic revolver oligos. Figure 5 shows plots of arrest rate versus mismatch rate for individual instances (technical triplicates for revolver oligos) of the four RT enzymes. The results are colour-coded to allow visual distinction of single instances of m^1^G versus m^2^_2_G. A visual inspection clearly shows, that the oligo-derived signals allow clear distinction between m^1^G versus m^2^_2_G in the identical sequence context of the revolver assay. In contrast, signals from native tRNAs only show a general trend of m^2^_2_G to higher mismatch and arrest rates (see also Supplement Figure S8B), but lack clear separation of the instances from m^1^G instances, thereby providing a plausible hypothesis, why the machine learning approach might be impractical using only native tRNA.

## DISCUSSION

The central objective of this work concerns the performance of machine learning approaches for automated modification calling. We advocate the use of e.g. a random forest model as an unbiased entity, which determines threshold values without human input, yet leaves the possibility open to read out this parameter *a posteriori*. Here, we compared 13 commercially available reverse transcriptases of different origin, based on their individual behaviour at m^1^A sites in tRNA of *S. cerevisiae*. The presented data show large variance of RT-signatures, illustrated in the differently expressed features, arrest and mismatch. In addition, some of the reverse transcriptases show an increased susceptibility to deletions at modified sites, termed as jumps, which we used to extend the RT-signature feature set. We taught these characteristics to a random forest model for supervised prediction and identification. In addition, to compare our selected RTs with TGIRT and HIV-RT, used in other m^1^A detection studies, we retrospectively collected data about their m^1^A dependent RT-signatures and their application in machine learning models. Interestingly we found strong correlations between the different RT-signature characteristics and the random forest performance. Especially, higher read-through, reached by increased mismatch and jump rates led to higher performance. *Vice versa*, increased arrest rates led to impeded distinguishability and thus detectability. Inadvertent arrest, occurring due to other modifications, RNA degradation or secondary and tertiary RNA structures, may introduce a strong background noise, thus complicating and possibly even hinder a prediction. Indeed, especially in order to distinguish modifications from each other, a test setup should be chosen aiming utmost diverse RT signatures and therefore providing signatures with the lowest possible arrest rates. Despite highly significant improvement in the AUC values, from previously 0.94 (22) to now 0.9964, the sobering truth is, that in the present state, the signal quality does not allow meaningful mapping of eukaryotic transcriptomes, which contain in the order of 10^7^ adenosines. The problem is compounded by the fact, that a significant number of m^1^A sites could plausible be modified sub-stoichiometrically, which would diminish signal strength. The only dataset featuring a potentially sufficient statistical basis would be that used by Grozhik *et al.* (29,30), who, significantly, conclude a very low number of m^1^A occurrences in human mRNAs. In further experiments, we then applied the three best performing RTs on other purine modifications with chemical alterations on their Watson–Crick face, very likely impeding cDNA synthesis, to evaluate the hypothesis, that, in general, every modification can be distinguished and detected. Indeed, our data on m^1^G and m^2^_2_G instances suggests that the apparent limitations are most likely due to coverage at analysed modified sites and that with an optimized experimental setup, e.g. selection of the reverse transcriptase and sufficient sequencing output, the modifications can be predicted and differentiated. Our study also indicates that synthetic oligonucleotides provide clearer RT-signatures compared to the more variegated patterns from native RNA, which has to be considered. Taking all these findings into account we could show that a prospective large-scale modification prediction depends on a carefully selected workflow, including an optimized experimental setup and unbiased, comparable data evaluation. By considering that the distinctness of RT-signatures is the major key for a strong and successful prediction, thorough RT screening and reaction setup for specific pattern generation at modified sites will help for further development of modification calling pipelines.

## ABBREVIATIONS

A selected list of abbreviations is available in the Supplementary Data.

## DATA AVAILABILITY

The bioinformatics modules, used for this study and implemented in Galaxy (46), are available in the GitHub repository (https://github.com/HelmGroup/Galaxy_modification_calling).

## Supplementary Material

gkaa113_Supplemental_File
