# Supplementary material for: Machine learning of reverse transcription signatures of variegated polymerases allows mapping and discrimination of methylated purines in limited transcriptomes
Source: Nucleic Acids Res. 2020 Feb 25;48(7):3734–46. doi: 10.1093/nar/gkaa113 (PMC7144921; doi:10.1093/nar/gkaa113)
Supplement: gkaa113_Supplemental_File [file gkaa113_supplemental_file.pdf]

Supplement

List of abbreviations (selected)

AUC	Area Under the (ROC) Curve
FN	False Negatives
FP	False Positives
FPR	False Positive Rate
MCC	Matthews Correlation Coefficient
PCC	Pearson Correlation Coefficient
PPV	Positive Predictive Value
RF	Random Forest
ROC	Receiver Operating Characteristic
RT	Reverse Transcription/Transcriptase
SVM	Support-Vector Machine
TN	True Negatives
TNR	True Negative Rate
TP	True Positives
TPR	True Positive Rate

Tables

Table S1: *Oligonucleotides applied in this study*

Type	Oligonucleotide	Sequence
**RNA**	**m^1A revolver**	5'-CACUGUAA**m^1A**NCUAACUUAGC-3'
**RNA**	**m^6A revolver - A**	5'-CACUGUAA**m^6A**ACUAACUUAGC-3'
**RNA**	**m^6A revolver - C**	5'-CACUGUAA**m^6A**CCUAACUUAGC-3'
**RNA**	**m^6A revolver - G**	5'-CACUGUAA**m^6A**GCUAACUUAGC-3'
**RNA**	**m^6A revolver - U**	5'-CACUGUAA**m^6A**UCUAACUUAGC-3'
**RNA**	**m^6_2A revolver - A**	5'-CACUGUAA**m^6_2A**ACUAACUUAGC-3'
**RNA**	**m^6_2A revolver - C**	5'-CACUGUAA**m^6_2A**CCUAACUUAGC-3'
**RNA**	**m^6_2A revolver - G**	5'-CACUGUAA**m^6_2A**GCUAACUUAGC-3'
**RNA**	**m^6_2A revolver - U**	5'-CACUGUAA**m^6_2A**UCUAACUUAGC-3'
**RNA**	**m^1G revolver**	5'-CACUGUAA**m^1G**NCUAACUUAGC-3'
**RNA**	**m^2_2G revolver**	5'-CACUGUAA**m^2_2G**NCUAACUUAGC-3'
**DNA**	**3'-adapter**	5'-(P)-CNNNNNNNNNAGATCGGAAGAGCGTCGTGTAGGGAAAGAGTGT-3'-C6-spacer
**DNA**	**RT primer**	5'-ACACTCTTTCCCTACACGACGCTCTTCCGATCT-3'
**DNA**	**5'-adapter strand 1**	5'-GTGACTGGAGTTCAGACGTGTGCTCTTCCGATCTGG-3'
**DNA**	**5'-adapter strand 2**	5'-GTGACTGGAGTTCAGACGTGTGCTCTTCCGATCTTT-3'
**DNA**	**5'-adapter strand 3**	5'-(P)-AGATCGGAAGAGCACACGTCTGAACTCCAGTCAC-3'-C6-spacer
**DNA**	**P7 PCR primer**	5'-CAAGCAGAAGACGGCATACGAGAT77777777GTGACTGGAGTTCAGACGTGTGC-TCTTCCGATCT-3'
**DNA**	**P5 PCR primer**	5'-AATGATACGGCGACCACCGAGATCTACAC55555555ACACTCTTTCCCTACACGA-CGCTCTTCCGATCT-3'

(P) = phosphate. N = A, C, G, U/T. (7777777) and (55555555) constitute standard Illumina Nextera barcodes (N701-N712 and N501-N508).

The corresponding m^1A, m^1G and m^2_2G revolver oligo was used for experiments as synthesized, and the m^6A and m^6_2A revolver oligos were mixed in equal amounts respectively before use.

Table S2: *RT conditions*

	Reverse Transcriptase	Components (final concentration)
**#1**	**M-MuLV**	M-MuLV Buffer (1x), RT primer (5 µM), dNTP mix (0.5 mM), M-MuLV RT (10 U/µL)
**#2**	**AMV**	AMV Buffer (1x), RT primer (5 µM), dNTP mix (0.5 mM), AMV RT (10 U/µL)
**#3**	**ProtoScript® II**	ProtoScript® II Buffer (1x), RT primer (5 µM), dNTP mix (0.5 mM), DTT (10 mM), ProtoScript® II RT (10 U/µL)
**#4**	**GoScript™**	GoScript™ Buffer (1x), RT primer (5 µM), dNTP mix (0.5 mM), $MgCl_2$ (3 mM), GoScript™ RT (10 U/µL)
**#5**	**SuperScript™ III**	First Strand Buffer (1x), RT primer (5 µM), dNTP mix (0.5 mM), BSA (50 µg/mL), DTT (5 mM), SuperScript™ III RT (10 U/µL)
**#6**	**RevertAid™**	RevertAid™ Buffer (1x), RT primer (5 µM), dNTP mix (0.5 mM), RevertAid™ RT (10 U/µL)
**#7**	**AccuScript™**	AccuScript™ Buffer (1x), RT primer (5 µM), dNTP mix (0.5 mM), DTT (10 mM), AccuScript™ RT (10 U/µL)
**#8**	**AffinityScript™**	AffinityScript™ Buffer (1x), RT primer (5 µM), dNTP mix (0.5 mM), AffinityScript™ RT (10 U/µL)
**#9**	**M-MLV**	M-MLV Buffer (1x), RT primer (5 µM), dNTP mix (0.5 mM), M-MLV RT (10 U/µL)
**#10**	**MonsterScript™**	MonsterScript™ Buffer (1x), RT primer (5 µM), dNTP mix (0.5 mM), MonsterScript™ RT (2.5 U/µL)
**#11**	**EpiScript™**	EpiScript™ Buffer (1x), RT primer (5 µM), dNTP mix (0.5 mM), DTT (10 mM), EpiScript™ (5 U/µL)
**#12**	**SuperScript™ IV**	SuperScript™ IV Buffer (1x), RT primer (5 µM), dNTP mix (0.5 mM), DTT (5 mM), SuperScript™ IV RT (10 U/µL)
**#13**	**Volcano**	Volcano Buffer (1x), RT primer (5 µM), dNTP mix (0.5 mM), Volcano DNA polymerase (0.25 U/µL)

Reactions were performed at 45°C for 1h for RT #1 to #12, and at 60°C for 1h for RT #13.

Table S3: *RT machine learning statistics*

	Reverse Transcriptase	Sensitivity	Specificity	PPV	NPV	MCC	AUC
**#1**	**M-MuLV**	0.9661	0.9827	0.9818	0.9837	**0.9841**	**0.9826**
**#2**	**AMV**	0.9578	0.9783	0.9750	0.9816	**0.9821**	**0.9769**
**#3**	**ProtoScript® II**	0.9791	0.9892	0.9891	0.9894	**0.9899**	**0.9897**
**#4**	**GoScript™**	0.9582	0.9785	0.9749	0.9821	**0.9824**	**0.9770**
**#5**	**SuperScript™ III**	0.9763	0.9879	0.9865	0.9892	**0.9896**	**0.9872**
**#6**	**RevertAid™**	0.9761	0.9877	0.9876	0.9878	**0.9884**	**0.9884**
**#7**	**AccuScript™**	0.9707	0.9849	0.9876	0.9823	**0.9835**	**0.9879**
**#8**	**AffinityScript™**	0.9692	0.9843	0.9833	0.9853	**0.9858**	**0.9840**
**#9**	**M-MLV**	0.9547	0.9769	0.9752	0.9786	**0.9793**	**0.9764**
**#10**	**MonsterScript™**	0.9594	0.9792	0.9748	0.9836	**0.9836**	**0.9769**
**#11**	**EpiScript™**	0.9807	0.9901	0.9897	0.9906	**0.9909**	**0.9902**
**#12**	**SuperScript™ IV**	0.9904	0.9950	0.9962	0.9938	**0.9942**	**0.9964**
**#13**	**Volcano**	0.9734	0.9864	0.9869	0.9859	**0.9865**	**0.9875**
			**Pearson Correlation Coefficient (PCC)**			**0.9016**	

Data was averaged from triplicates. PPV = Positive Predictive Value. NPV = Negative Predictive Value. MCC = Matthews Correlation Coefficient. AUC = Area Under the ROC Curve (Receiver Operating Characteristic (ROC)). PCC = Pearson Correlation Coefficient.

Table S4: *Feature Importance*

	Reverse Transcriptase	Arrest [%]	Mismatch [%]	C [%]	G [%]	T [%]	Jump [%]
**#1**	**M-MuLV**	16.346	15.583	4.505	8.670	2.495	10.839
**#2**	**AMV**	19.061	14.667	4.523	10.257	5.032	5.345
**#3**	**ProtoScript® II**	18.253	16.933	3.301	7.207	3.831	10.225
**#4**	**GoScript™**	21.359	14.259	3.810	7.435	4.346	3.845
**#5**	**SuperScript™ III**	18.760	17.697	2.823	5.156	4.374	9.448
**#6**	**RevertAid™**	15.519	17.415	3.185	6.278	3.607	12.218
**#7**	**AccuScript™**	16.197	14.832	3.628	8.121	3.875	11.685
**#8**	**AffinityScript™**	13.264	17.975	3.260	7.326	4.568	13.745
**#9**	**M-MLV**	17.981	14.201	4.548	7.283	4.170	8.473
**#10**	**MonsterScript™**	23.160	14.077	4.282	6.957	4.105	6.473
**#11**	**EpiScript™**	15.952	17.112	3.467	8.063	3.950	11.499
**#12**	**SuperScript™ IV**	8.061	20.141	3.672	8.058	6.364	16.020
**#13**	**Volcano**	16.543	15.273	6.002	8.070	3.339	7.850

Data was averaged from triplicates. Jump = jump rate. C, T, G = mismatch components, which add up to 100 %. Mismatch = mismatch rate. Arrest = arrest rate. Percentages represent feature importance in random forest analysis = mean loss in classification accuracy, if values of respective feature are permutated.

Table S5: *Machine Learning m^1G/m^2_2G vs. other Guanosines (RF #1)*

	Reverse Transcriptase	Training instances (from 2 datasets) after coverage filtering (>20) m^1G / m^2_2G	Prediction performance m^1G & m^2_2G AUC (training) RF #1	Test instances (from 1 dataset) after coverage filtering (>20) m^1G / m^2_2G	Guanosines classified as modified (from 988 guanosines) in RF #1	Correct classified m^1G test instances in RF #1	Correct classified m^2_2G test instances in RF #1
**#3**	**ProtoScript® II**	27 / 23	0.9641	11 / 8	62	10	6
**#5**	**SuperScript™ III**	31 / 33	0.9727	16 / 19	81	12	18
**#11**	**EpiScript™**	22 / 20	0.9439	16 / 14	109	12	14
**#12**	**SuperScript™ IV**	32 / 38	0.9801	16 / 22	67	13	20

Table S6: *Machine Learning m^1G vs. m^2_2G (RF #2) – m^1G prediction*

	Reverse Transcriptase	Training instances (from 2 datasets) after coverage filtering (>20) m^1G	Prediction performance m^1G AUC (training) RF #2	Test dataset (Guanosines classified as modified in RF #1)	As m^1G classified instances in test dataset in RF #2	Test instances (from RF #1) after coverage filtering (>20) m^1G	Correct classified m^1G test instances in RF #2
**#3**	**ProtoScript® II**	27	0.9226	62	45	10	10
**#5**	**SuperScript™ III**	31	0.9605	81	37	12	12
**#11**	**EpiScript™**	22	0.9606	109	65	12	9
**#12**	**SuperScript™ IV**	32	0.9647	67	35	13	13

Table S7: *Machine Learning m^1G vs. m^2_2G (RF #2) – m^2_2G prediction*

	Reverse Transcriptase	Training instances (from 2 datasets) after coverage filtering (>20) m^2_2G	Prediction performance m^2_2G AUC (training) RF #2	Test dataset (Guanosines classified as modified in RF #1)	As m^2_2G classified instances in test dataset in RF #2	Test instances (from RF #1) after coverage filtering (>20) m^2_2G	Correct classified m^2_2G test instances in RF #2
**#3**	**ProtoScript® II**	23	0.9596	62	16	6	5
**#5**	**SuperScript™ III**	33	0.9797	81	28	18	18
**#11**	**EpiScript™**	20	0.9433	109	40	14	10
**#12**	**SuperScript™ IV**	38	0.9820	67	32	20	20

Table S8: *m^1G and m^2_2G in yeast total tRNA*

	Reverse Transcriptase	m^1G Instances (Triplicates)	m^2_2G Instances (Triplicates)
**#3**	**ProtoScript® II**	12	6
**#5**	**SuperScript™ III**	14	14
**#11**	**EpiScript™**	9	5
**#12**	**SuperScript™ IV**	15	17

Listed and used for comparison in *Figure 5* and *Supplement Figure S8* are m^1G and m^2_2G sites which are present in all 3 total tRNA replicates and show a coverage of at least 20 reads in at least 2 replicates.

Table S9: *Synthetic m^1A revolver oligonucleotides*

Type	Oligonucleotide (gene name)	Sequence
**RNA**	**MALAT1**	5’- GGUUUCCAGGACGGGGUUCA**m^1A**NUCCCUGCGGCGUCUUUGCU -3’
**RNA**	**C9orf100**	5‘-GACACUGCUAGCUGGGUUCA**m^1A**NUCCCAGCUCCAGCAGUUGC -3‘
**RNA**	**PRUNE**	5‘-UUCGCCGUGUGGCGGGUUCG**m^1A**NUCCCGCCUCCUGACUCUGG -3‘
**RNA**	**ZNF664**	5‘-AGGCGUUCAGUCAGAGUUCG**m^1A**NCCUCUGCAUCCACCAGAGA -3‘
**RNA**	**GTF3C2**	5‘-GGGCAGUCAGGGCUGGUUCG**m^1A**NUCCAUUUUGUCCGUGGACU -3‘
**RNA**	**BRD2**	5‘-GCACCAGGGAAGAGGAUUCG**m^1A**NAACCCUCUCUCUUGUAUGA -3‘
**RNA**	**ATAD3B**	5‘-CAAGCUCUUUGACUGGGCCA**m^1A**NACCAGCCGGCGCGGCCUCC -3‘
**RNA**	**TP53I13**	5‘-CAGGGGGCUGUGUCUGUUCA**m^1A**NUCAGGCUUCCCCGGCCCCU -3‘
**RNA**	**SRSF1**	5‘-CGAGGCGGCGGCGGGGGUGG**m^1A**NGUGGCGGAGCUCCCCGAGG -3‘
**RNA**	**UBC**	5‘-CGUCUCAGAGGUGGGAUGCA**m^1A**NUCUUCGUGAAGACACUCAC -3‘
**RNA**	**ND5**	5‘-AACCCCAUUAAACGCCUGGC**m^1A**NCCGGAAGCCUAUUCGCAGG -3‘
**RNA**	**COX1**	5’-AGUAGAAGAACCCUCCAUAA**m^1A**NCUGGAGUGACUAUAUGGAU-3’
**RNA**	**COX2**	5’-CCCUCCCUUACCAUCAAAUC**m^1A**NUUGGCCACCAAUGGUACUG-3’
**RNA**	**COX3**	5’-CAAACAUCACUUUGGCUUCG**m^1A**NGCCGCCGCCUGAUACUGGC-3’

Synthetic oligonucleotides (40 nt) with internal m^1A at position 21 and degenerated +1 position. Sequences were chosen according to reported m^1A sites in cytosolic and mitochondrial RNA from Safra *et al.* ***(2)*** and *were* used for machine learning in *Supplement Table S10.*

Table S10: *RT machine learning statistics (extended dataset)*

	Reverse Transcriptase	Sensitivity	Specificity	PPV	NPV	MCC	AUC
**#3**	**ProtoScript® II**	0.9938	0.9900	0.9893	0.9937	0.9834	0.9915
**#11**	**EpiScript™**	0.9910	0.9790	0.9777	0.9909	0.9693	0.9843
**#12**	**SuperScript™ IV**	0.9922	0.9951	0.9950	0.9917	0.9870	0.9933

Training datasets of 3 RTs for machine learning were extended with instances from a sequencing run with 14 synthetic m^1A oligonucleotides, degenerated at the +1 position (see *Supplement Table S9*). Trained random forest models from 3 different RTs were tested and applied on yeast tRNA. PPV = Positive Predictive Value. NPV = Negative Predictive Value. MCC = Matthews Correlation Coefficient. AUC = Area Under the ROC Curve (Receiver Operating Characteristic (ROC)). PCC = Pearson Correlation Coefficient.

Figures

Figure S1

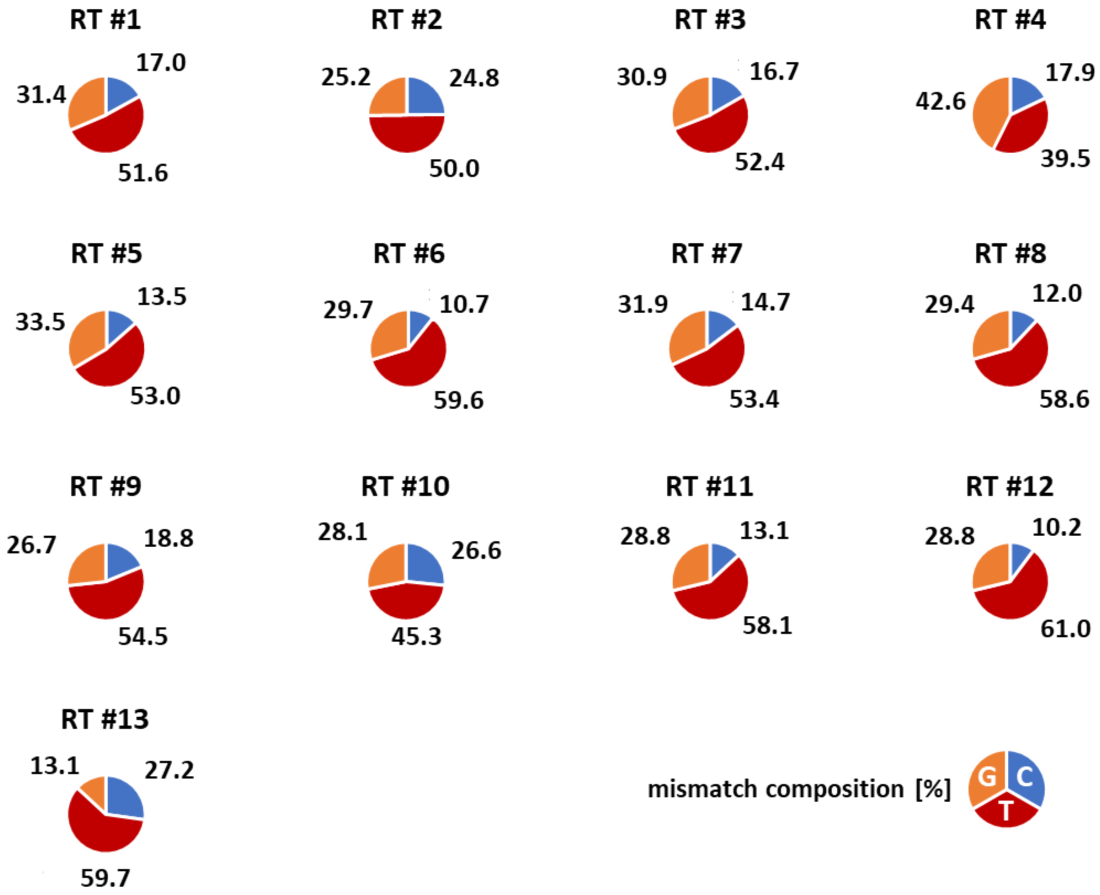

Figure S1: *Average mismatch composition by RT. Shows, for each RT, the C-mismatch (blue), G-mismatch (orange) and T-mismatch (red) averages from 3 individual library preparation and sequencing runs (triplicates) of total tRNA from Saccharomyces cerevisiae as percentage of the overall mismatch (100%) at* m^1*A-sites. (See also Supplement Figure S9 for additional information on TGIRT and HIV-RT)*

Figure S2

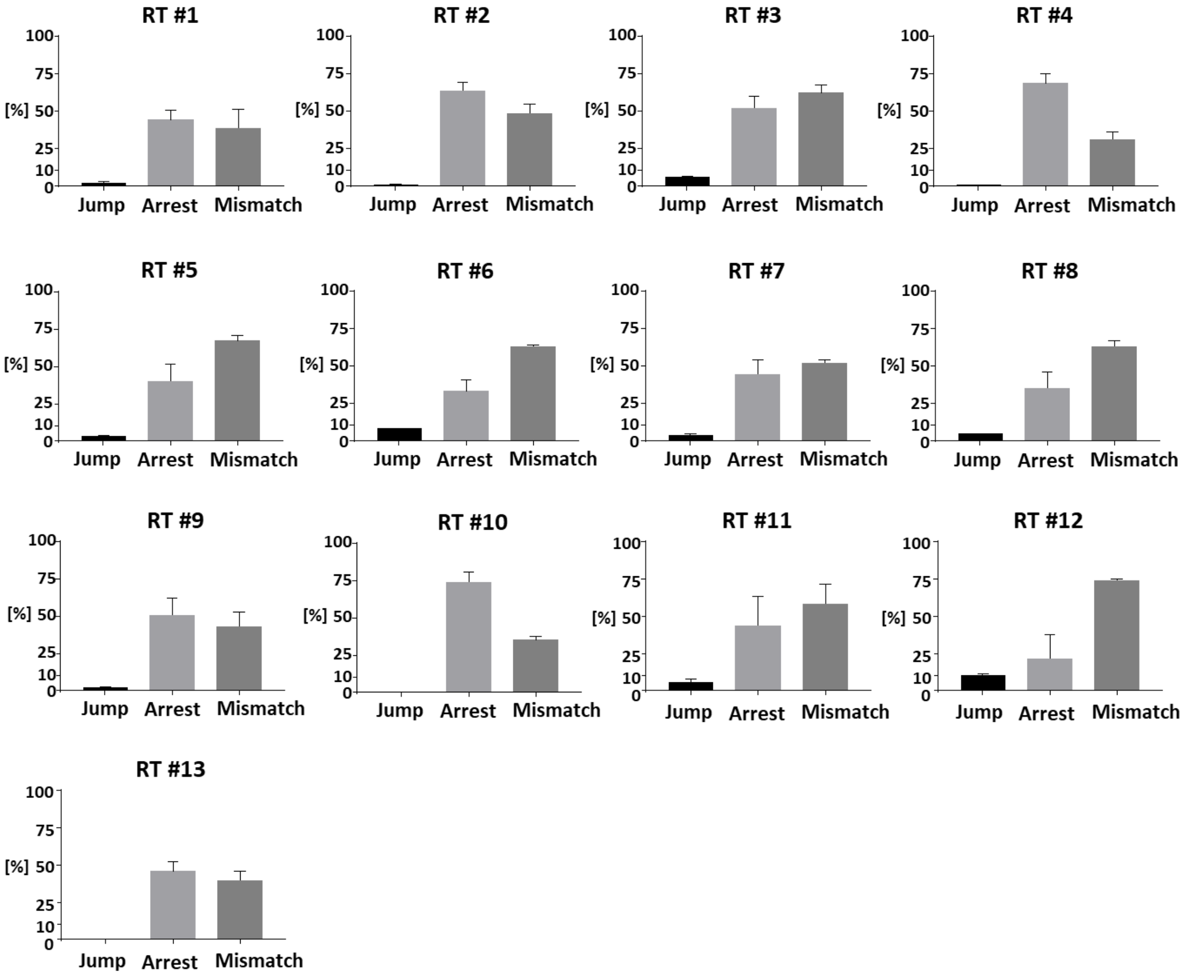

Figure S2: *RT-signature features by RT. Shows, for each RT, the average jump, arrest and mismatch rates from 3 individual library preparation and sequencing runs (triplicates) of total tRNA from Saccharomyces cerevisiae as percentage at* m^1A*-sites. (black bars indicate standard deviations across 3 sequencing runs). Arrest rate percentages refer to the reads covering the 3' adjacent position of* m^1A *(+1 position). Mismatch and jump rate percentages refer to the reads covering the* m^1A *position. (See also Supplement Figure S9 for additional information on TGIRT and HIV-RT)*

Figure S3

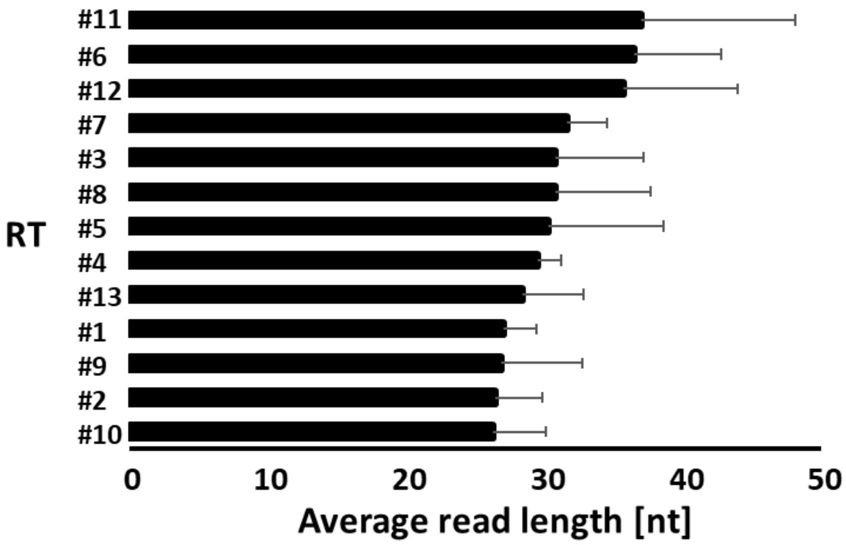

Figure S3: *Average read length by RT. Shows, for each RT, the average read length from 3 individual library preparation and sequencing runs (triplicates) of total tRNA Saccharomyces cerevisiae (black bars indicate standard deviations across 3 sequencing runs). (See also Supplement Figure S9 for additional information on TGIRT and HIV-RT)*

Figure S4

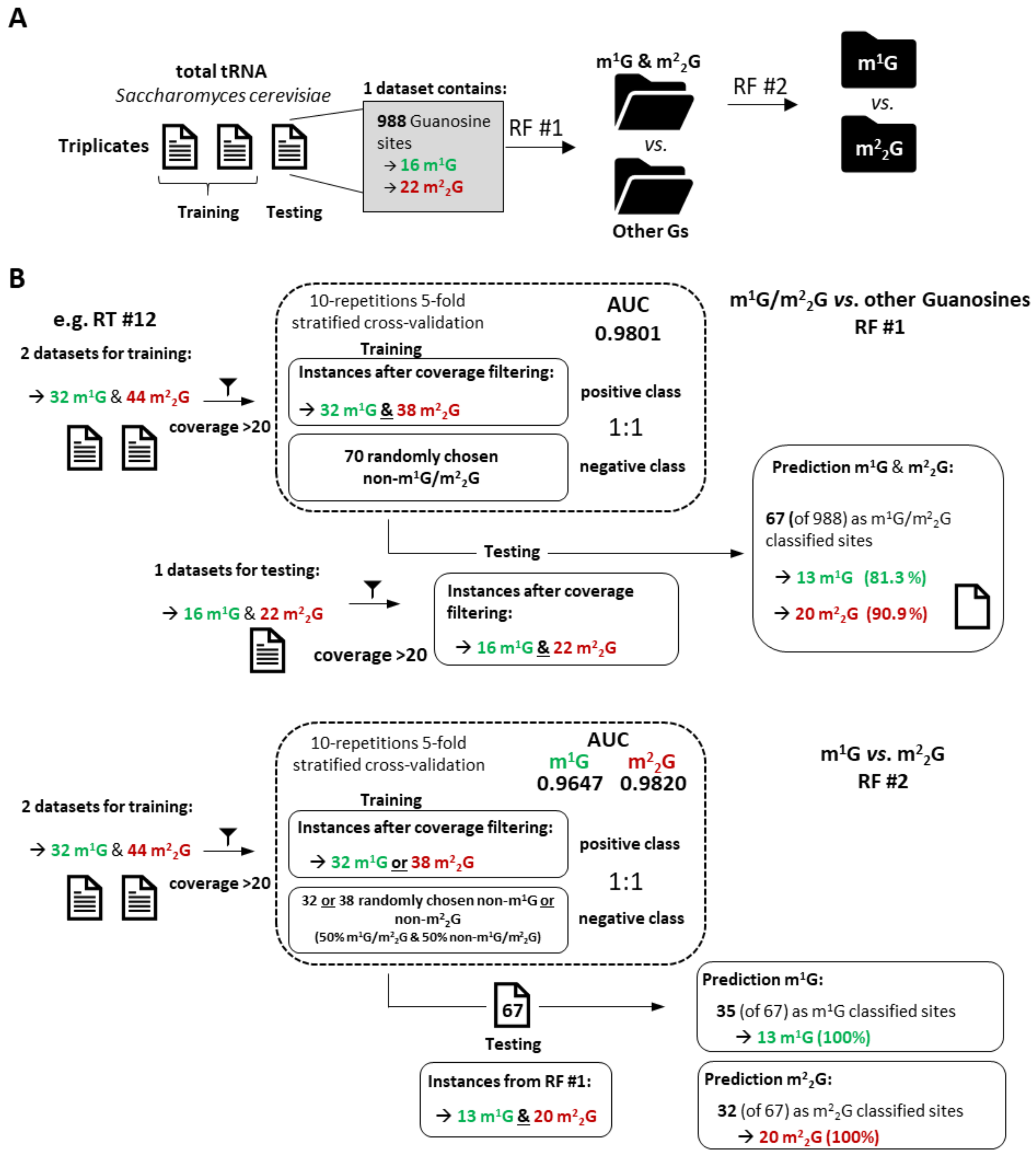
A
total tRNA
Saccharomyces cerevisiae
Triplicates
Training
Testing
1 dataset contains:
988 Guanosine sites
→ 16 m1G
→ 22 m22G
RF #1
m1G & m22G
vs.
Other Gs
RF #2
m1G
vs.
m22G
B
e.g. RT #12
2 datasets for training:
→ 32 m1G & 44 m22G
coverage >20
10-repetitions 5-fold stratified cross-validation
AUC 0.9801
Training
Instances after coverage filtering:
→ 32 m1G & 38 m22G
positive class
1:1
70 randomly chosen non-m1G/m22G
negative class
m1G/m22G vs. other Guanosines
RF #1
Testing
1 datasets for testing:
→ 16 m1G & 22 m22G
coverage >20
Instances after coverage filtering:
→ 16 m1G & 22 m22G
Prediction m1G & m22G:
67 (of 988) as m1G/m22G classified sites
→ 13 m1G (81.3 %)
→ 20 m22G (90.9 %)
2 datasets for training:
→ 32 m1G & 44 m22G
coverage >20
10-repetitions 5-fold stratified cross-validation
AUC
m1G 0.9647
m22G 0.9820
Training
Instances after coverage filtering:
→ 32 m1G or 38 m22G
positive class
1:1
32 or 38 randomly chosen non-m1G or non-m22G
(50% m1G/m22G & 50% non-m1G/m22G)
negative class
m1G vs. m22G
RF #2
67
Testing
Instances from RF #1:
→ 13 m1G & 20 m22G
Prediction m1G:
35 (of 67) as m1G classified sites
→ 13 m1G (100%)
Prediction m22G:
32 (of 67) as m22G classified sites
→ 20 m22G (100%)

Figure S4: *(**A**) General procedure. Three replicates of total tRNA from Saccharomyces cerevisiae were prepared for sequencing. For each RT two replicate datasets were used for random forest training and the third dataset for testing. Each replicate dataset contains 988 guanosine sites, including 16 m^1G and 22 m^2_2G sites. In a first RF model (RF #1) we trained with m^1G and m^2_2G sites and tested for joint m^1G and m^2_2G separation from other guanosines. In a second RF model (RF #2) the modified sites from RF #1 were separated in m^1G and m^2_2G sites, therefore two independent RF models were trained on m^1G and m^2_2G respectively, with the respective other modification as negative class. (**B**) RT #12 prediction performance. In a first random forest model (m^1G/m^2_2G vs. other Guanosines – RF #1) we separated m^1G together with m^2_2G sites from other guanosines. From the two training datasets for RT #12 with corresponding 32 m^1G (2x16) and 44 m^2_2G (2x22) sites, after removal of the low-coverage instances (coverage <20) 32 m^1G and 38 m^2_2G sites remained in the positive class for training. The negative class contained 70 randomly chosen non-m^1G/m^2_2G sites. The trained model (AUC value of 0.9801) was then tested on the third remaining dataset with corresponding 16 m^1G, 22 m^2_2G after removal of the low-coverage instances (coverage <20) and 986 other guanosine sites. The model classified 67 guanosine sites as m^1G and m^2_2G, including 13 m^1G (81.3 %) and 20 m^2_2G (90.9 %). These 67 instances were then used as test dataset in a second RF model (m^1G vs. m^2_2G – RF #2). Therefore, two independent RF models were trained on m^1G (32 sites and an AUC of 0.9647) and m^2_2G (38 sites and an AUC of 0.9820) respectively, with the respective other modification together with non-modified guanosines as negative class (1:1 composition). The separated prediction of m^1G and m^2_2G strongly decreased the number of false positive instances, wherein the selection of m^2_2G generally worked better. The m^1G prediction classified 35 sites as m^1G, including 13 correct classified m^1G (100 % correct classified m^1G instances) and the m^2_2G prediction identified 32 sites as m^2_2G, including 20 correct classified m^2_2G (100 % correct classified m^2_2G instances).*

Figure S5

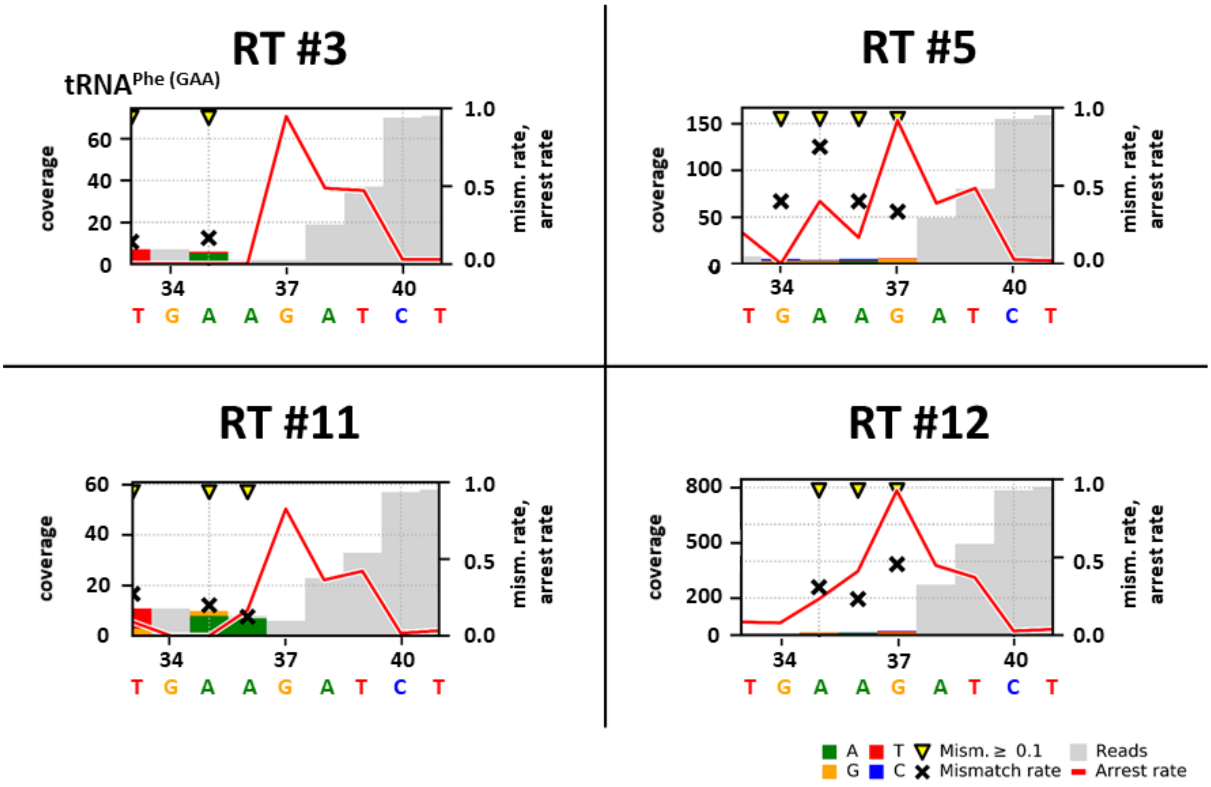

Figure S5: *Examples for RT-signatures of wybutosine by RT. Graphs from the wybutosine site at position 37 in tRNA$^{Phe\ (GAA)}$ from Saccharomyces cerevisiae are shown. Sites with error rates of more than 10% are highlighted with yellow arrows. Colored bars indicate the nature of the reads. The mismatch rate is depicted as black cross and the arrest rate as red line. Note that statements on average values stated in the text may differ from these individual signatures.*

Figure S6

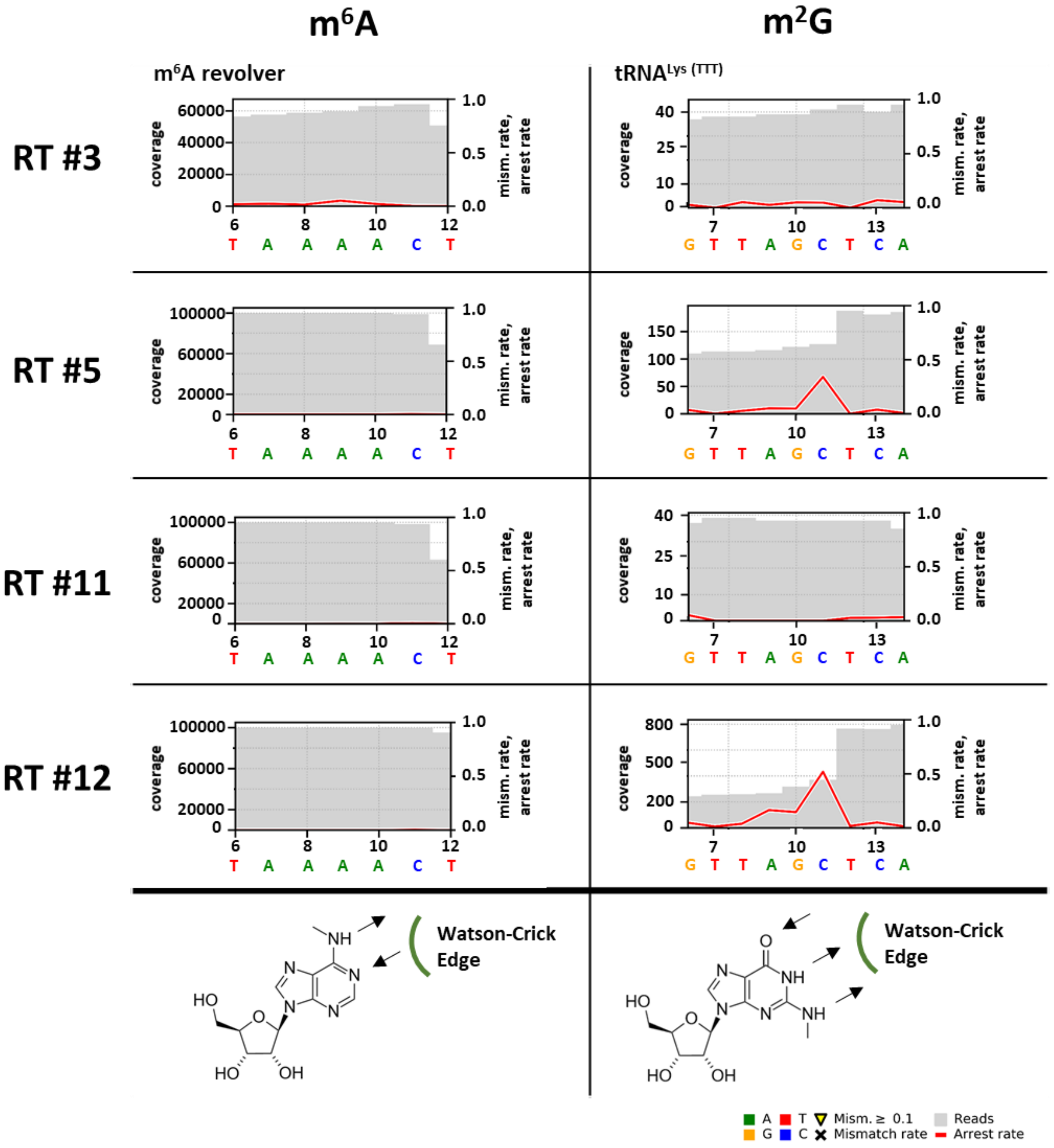

Figure S6: *Examples for RT-signatures of m⁶A and m²G by RT and the expected Watson-Crick base-pairing. For m⁶A, graphs from the revolver oligo with a neighboring A, 3' adjacent (+1 position) to the modified site at position 9, are shown. For m²G, graphs from an m²G site at position 10 in tRNA$^{Lys\ (TTT)}$ from Saccharomyces cerevisiae are shown. Sites with error rates of more than 10% are highlighted with yellow arrows. Colored bars indicate the nature of the reads. The mismatch rate is depicted as black cross and the arrest rate as red line. Note that statements on average values stated in the text may differ from these individual signatures.*

Figure S7

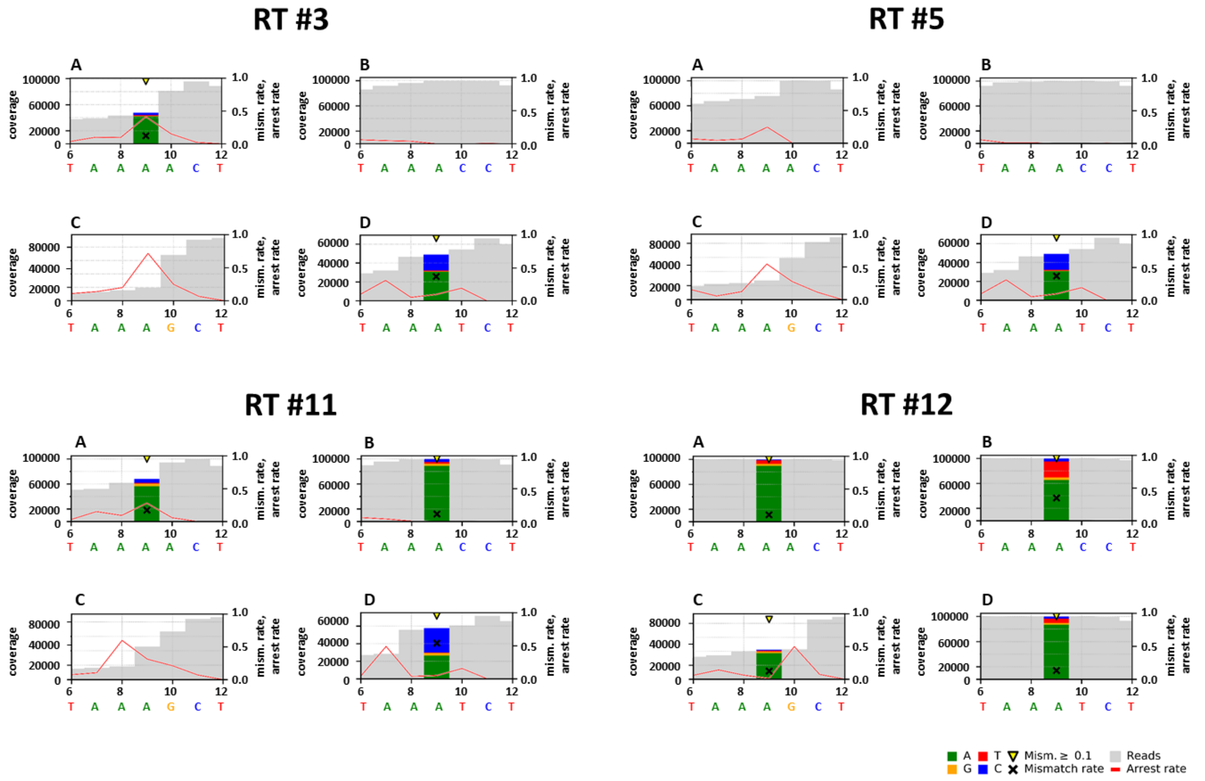

Figure S7: *RT-signatures of m$^6_2$A by RT from revolver oligo analysis. Graphs from the revolver oligo with a neighboring A (**A**), C (**B**), G (**C**) and T (**D**) 3' adjacent (+1 position) to the modified site at position 9, are shown. Sites with error rates of more than 10% are highlighted with yellow arrows. Colored bars indicate the nature of the reads. The mismatch rate is depicted as black cross and the arrest rate as red line. The modified site is shown at position 9 in the middle of the considered sequence.*

Figure S8

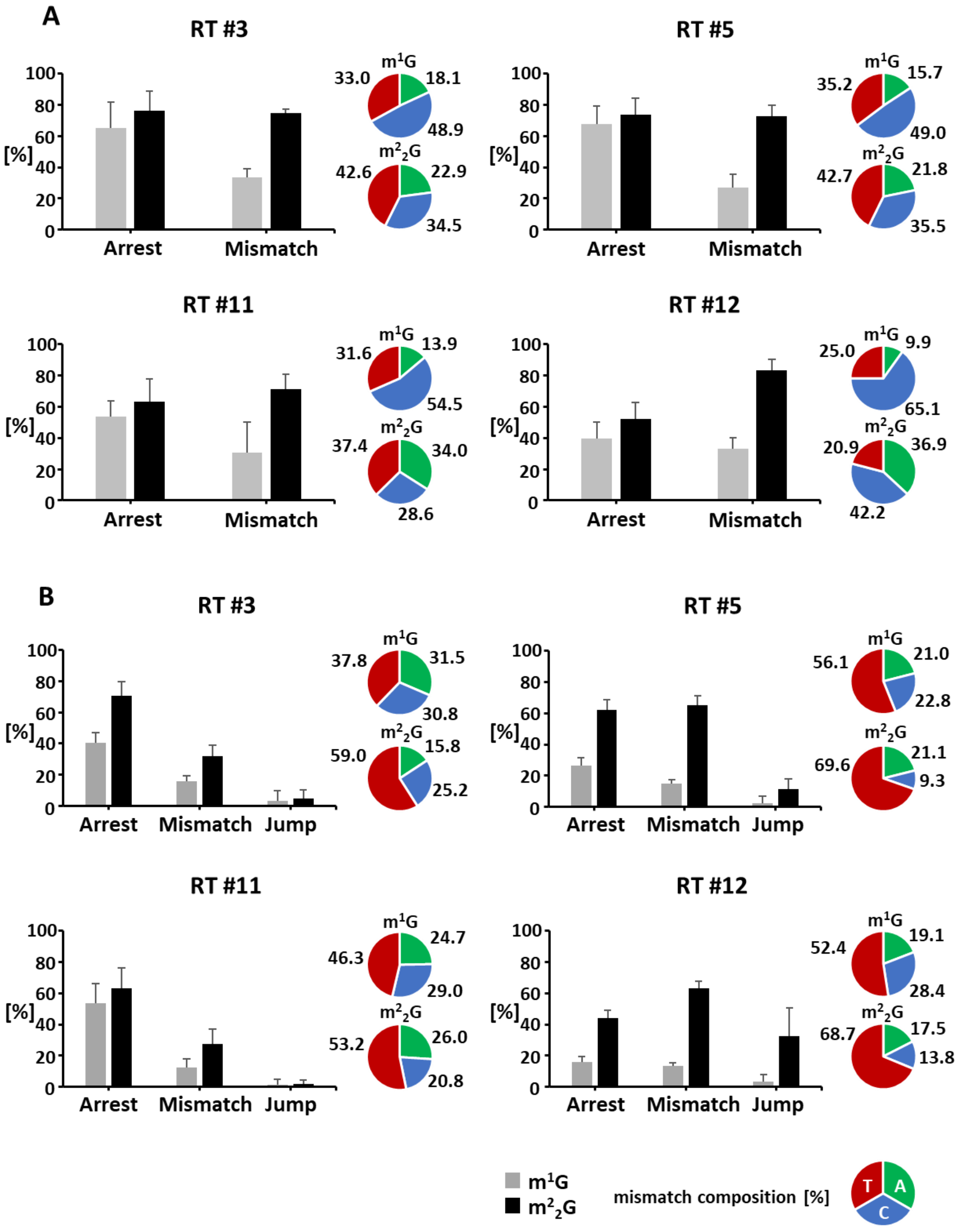

Figure S8: *RT-signature overview at m^{1}G and m$^2_2$G sites in synthetic revolver oligos (**A**) and total tRNA from Saccharomyces cerevisiae (**B**) by RT. Shows, for each RT and 3 individual library preparation and sequencing runs (triplicates), a bar plot with the average arrest and mismatch rate (and the average jump rate for (**B**)) at m^{1}G- (grey) and m$^2_2$G-sites (black) as percentage (black bars indicate standard deviations across 3 sequencing runs) and pie charts with the individual mismatch composition as average percentage of the overall mismatch (100 %) at m^{1}G- and m$^2_2$G-sites, including A-mismatch (green), C-mismatch (blue) and T-mismatch (red). Data from m^{1}G and m$^2_2$G sites which are present in all 3 total tRNA replicates and show a coverage of at least 20 reads in at least 2 replicates were considered (see Supplement Table S8 for more details**).** In general, arrest rate percentages refer to the reads covering the 3' adjacent position of m^{1}G/m$^2_2$G (+1 position). Mismatch and jump rate, as well as mismatch composition percentages refer to the reads covering the m^{1}G/m$^2_2$G position.*

Figure S9

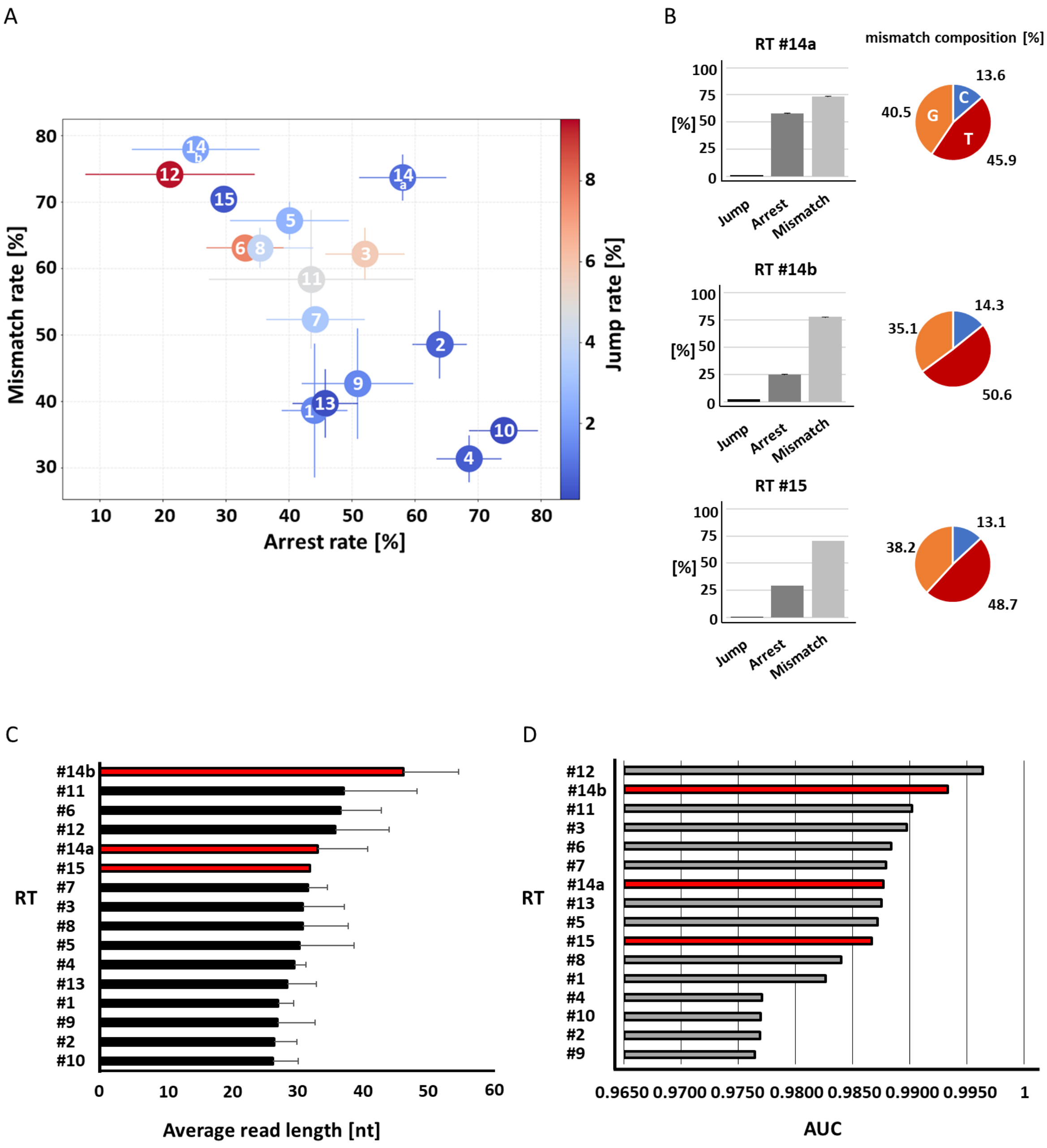
A
B
RT #14a
mismatch composition [%]
RT #14b
RT #15
Jump
Arrest
Mismatch
13.6
40.5
45.9
14.3
35.1
50.6
13.1
38.2
48.7
Mismatch rate [%]
Arrest rate [%]
Jump rate [%]
C
D
RT
Average read length [nt]
AUC

Figure S9: *(A) Scatter plot showing the m^1A signatures of the 13 RTs, TGIRT (RT #14a and #14b) and wildtype HIV-RT (RT# 15) at 26 m^1A sites in yeast cytosolic tRNA. Data for the 13 RTs and TGIRT are averaged from 3 sequencing runs, i.e. triplicates. Error bars show standard deviations of arrest and mismatch rates across 3 sequencing runs. Data for the wildtype HIV-RT (RT# 15) derives from a single replicate. The colour-code represents the jump rate. Arrest rate percentages refer to the reads covering the 3' adjacent position of m^1A (+1 position). Mismatch and jump rate percentages refer to the reads covering the m^1A position. (B) RT-signature features and mismatch composition by RT. Shows, for TGIRT (RT #14a and #14b), the average jump, arrest, and mismatch rates, as well as the C-mismatch (blue), G-mismatch (orange) and T-mismatch (red) averages as percentage of the overall mismatch (100%) from 3 individual library preparation and sequencing runs (triplicates) of total tRNA from Saccharomyces cerevisiae as percentage at m^1A-sites. (black bars indicate standard deviations across 3 sequencing runs). Data for the wildtype HIV-RT (RT #15) derives from a single replicate. Arrest rate percentages refer to the reads covering the 3' adjacent position of m^1A (+1 position). Mismatch and jump rate percentages refer to the reads covering the m^1A position. (C) Average read length by RT. Shows, for the 13 RTs and TGIRT, the average read length from 3 individual library preparation and sequencing runs (triplicates) of total tRNA from Saccharomyces cerevisiae (black bars indicate standard deviations across 3 sequencing runs). Data for the wildtype HIV-RT (RT #15) derives from a single replicate. (D) Random Forest performance (AUC) by RT. Shows, for each RT (except wildtype HIV-RT (RT #15)), the average AUC from 3 individual library preparation and sequencing runs (triplicates) of total tRNA Saccharomyces cerevisiae. Data for the wildtype HIV-RT (RT #15) derives from a single replicate*

Library preparation (TGIRT and wildtype HIV-RT)

TGIRT™ (RT #14) (Cat. No. TGIRT50, InGex, LLC) and wildtype HIV-RT (RT #15) (from Guillaume Bec and Eric Ennifar, CNRS Strasbourg) were subjected to the same library preparation and analysis workflow, described in the Material and Methods part of this manuscript. In the case of TGIRT an adapted buffer composition (RT #14b) (as applied in Li *et al.* **(1)**) was tested in parallel to the standard conditions (RT #14a) (as applied in Safra *et al.* **(2)**). The RT conditions were as follows:

	**Reverse Transcriptase**	**Components (final concentration)**
**RT #14a**	**TGIRT™**	TGIRT Buffer (1x - 450 mM NaCl, 5 mM $MgCl_2$, 20 mM Tris-HCl, pH 7.5), RT primer (5 µM), dNTP mix (0.5 mM), DTT (5 mM), TGIRT (500 nM)
**RT #14b**	**TGIRT™**	TGIRT Buffer adapted (1x - 75 mM KCl, 3 mM $MgCl_2$, 50 mM Tris-HCl, pH 8.3), RT primer (5 µM), dNTP mix (0.5 mM), DTT (5 mM), TGIRT (500 nM)
**RT #15**	**HIV (wildtype)**	HIV-RT Buffer (1x - 75 mM KCl, 3 mM $MgCl_2$, 50 mM Tris-HCl, pH 8.3), dNTP mix (0.5 mM), DTT (5 mM), HIV-RT (0.5 U/µL)

Reactions were performed at 60°C for 1h for RT #14a, at 57°C for 1h for RT #14b, and at 45°C for 1h for RT#15.

Figure S10

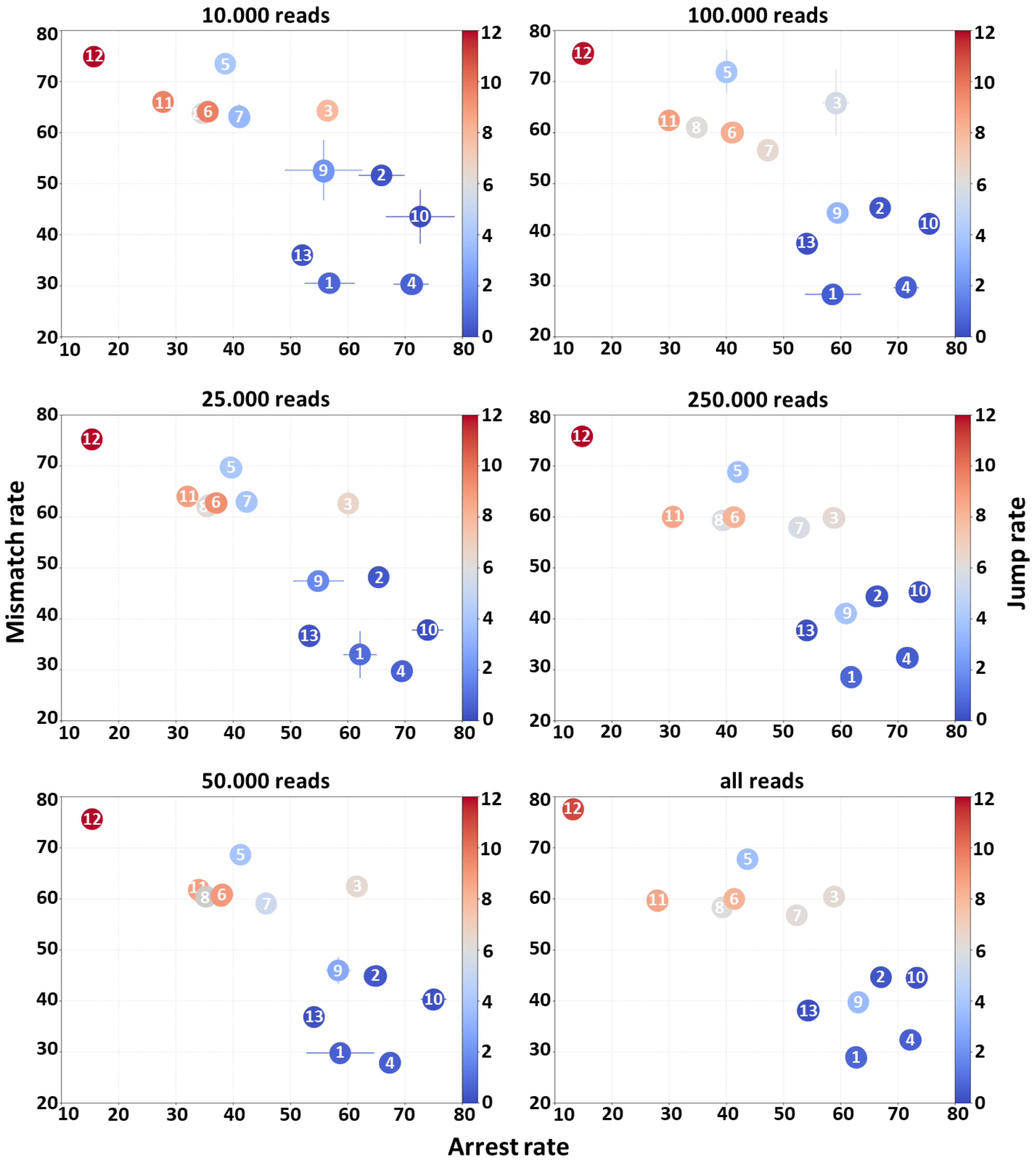

Figure S10: *Scatter plots showing the m¹A signatures of the 13 RTs by sampling identical numbers of reads for each RT and comparison with the plot deriving from all available reads (unequal). Data points for the sampled plots are averaged percentages from 3 rounds of random read selection from one sequencing run for each RT, error bars show standard deviations of arrest and mismatch rates across the 3 selection rounds. The colour-code represents the jump rate. Arrest rate percentages refer to the reads covering the 3' adjacent position of m¹A (+1 position). Mismatch and jump rate percentages refer to the reads covering the m¹A position.*

References

1. Li, X., Xiong, X., Zhang, M., Wang, K., Chen, Y., Zhou, J., Mao, Y., Lv, J., Yi, D., Chen, X.W. *et al.* (2017) Base-Resolution Mapping Reveals Distinct m(1)A Methylome in Nuclear- and Mitochondrial-Encoded Transcripts. *Mol Cell*, **68**, 993-1005.
2. Safra, M., Sas-Chen, A., Nir, R., Winkler, R., Nachshon, A., Bar-Yaacov, D., Erlacher, M., Rossmanith, W., Stern-Ginossar, N. and Schwartz, S. (2017) The m1A landscape on cytosolic and mitochondrial mRNA at single-base resolution. *Nature*, **551**, 251-255.
3. Hauenschild, R., Tserovski, L., Schmid, K., Thuring, K., Winz, M.L., Sharma, S., Entian, K.D., Wacheul, L., Lafontaine, D.L., Anderson, J. *et al.* (2015) The reverse transcription signature of N-1-methyladenosine in RNA-Seq is sequence dependent. *Nucleic Acids Res*, **43**, 9950-9964.
